# IFN-**γ** is a direct driver of crypt hyperplasia in celiac disease

**DOI:** 10.1172/JCI194858

**Published:** 2025-08-19

**Authors:** Jorunn Stamnaes, Daniel Stray, M. Fleur du Pré, Louise F. Risnes, Alisa E. Dewan, Jakeer Shaik, Maria Stensland, Knut E.A. Lundin, Ludvig M. Sollid

**Affiliations:** 1 Norwegian Coeliac Disease Research Centre, Institute of Clinical Medicine, University of Oslo, Oslo, Norway.; 2 Proteomics Core Facility, University of Oslo and Oslo University Hospital, Oslo, Norway.; 3 Department of Immunology, and; 4 Department of Gastroenterology, Oslo University Hospital-Rikshospitalet, Oslo, Norway.

**Keywords:** Gastroenterology, Immunology, Inflammation, Adaptive immunity, Cytokines, Proteomics

## Abstract

Crypt hyperplasia is a key feature of celiac disease (CeD) and several other small intestinal inflammatory conditions. Analysis of the gut epithelial crypt zone by mass spectrometry–based tissue proteomics revealed a strong IFN-γ signal in active CeD. This signal, hallmarked by increased expression of MHC molecules, was paralleled by diminished expression of proteins associated with fatty acid metabolism. Crypt hyperplasia and the same proteomic changes were observed in WT mice administered IFN-γ. In mice with conditional KO of the IFN-γ receptor in gut epithelial cells, these signature morphological and proteomic changes were not induced with IFN-γ administration. IFN-γ was thus a driver of crypt hyperplasia in CeD by acting directly on crypt epithelial cells. The results are relevant to other enteropathies with involvement of IFN-γ.

## Abstract 2

Small intestine crypt hyperplasia, a celiac disease hallmark, is caused by interferon-γ acting directly on crypt epithelial cells.

## Graphical abstract

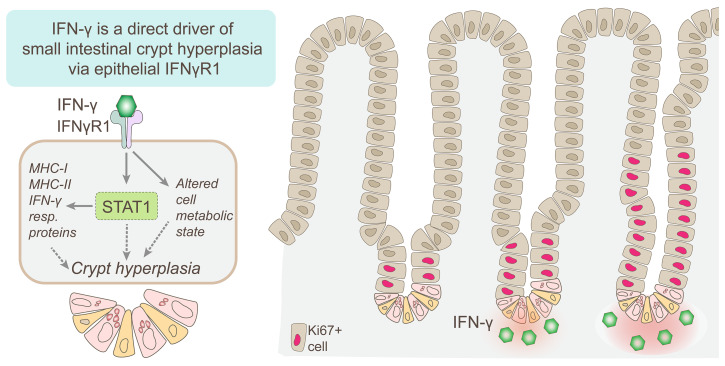

## Introduction

Crypt hyperplasia in the small intestine with elongation of the crypts of Lieberkühn is a pathological tissue alteration commonly seen in inflammatory conditions (1, 2). It is accompanied by increased epithelial cell turnover and also, when there is protracted inflammation, by blunting of the intestinal villi. In the intestine, intestinal stem cells (ISCs) positive for leucine-rich repeat containing G protein–coupled receptor 5 (Lgr5^+^) at the crypt base produce daughter cells that undergo proliferation in the transit-amplifying zone (3). The generated cells move along the crypt/villous axis and differentiate before they eventually are shed at the villous tip. Under normal physiological conditions in the small intestine of humans, the transit time from cell generation to cell expulsion is 3–5 days (4, 5). This transit process accelerates during inflammation, resulting in increased cell turnover. Reflecting the increased cell turnover in crypt hyperplasia, the number of proliferating cells is augmented, and the proliferating cells can be detected by staining for Ki67 or by labeling of newly synthesized DNA.

Perhaps the most notable condition hallmarked by crypt hyperplasia and villous blunting is celiac disease (CeD). This disease is a prevalent enteropathy caused by a maladapted immune response to cereal gluten proteins, and the condition is treated with a life-long strict gluten-free diet (6). The disease lesion is localized in the proximal small intestine, and the full-blown lesion has blunting of the villi (7), a 3-fold increase in proliferating cells per crypt (8), and the enterocyte turnover time is reduced to 24 hours (9). The drivers of the immune response to gluten in CeD are CD4^+^ T cells, which recognize deamidated gluten peptides in the context of disease-associated HLA-DQ allotypes (10). These CD4^+^ T cells are located in the lamina propria (11), which fits with the observation that intraepithelial CD4^+^ T cells are scarce in humans (12). The gluten-specific CD4^+^ T cells have a distinct phenotype (13) and a characteristic cytokine profile dominated by production of IFN-γ (14, 15). Intraepithelial CD8^+^ T cells also produce IFN-γ in active CeD (16, 17). Reflecting these observations, both proteomics and transcriptomics analyses of active celiac lesion tissue have revealed clear signatures of IFN-γ influence (18, 19). In line with the typical intestinal tissue remodeling of CeD, the diagnosis is conventionally made by histological examination of small intestinal biopsies with scoring according to the Marsh-Oberhuber scale (Marsh 0, 1, 2, 3a–c) (20). Marsh grades 2 and 3 both show the presence of crypt hyperplasia, whereas Marsh 3a-c grades in addition have increasing degrees of villous blunting and are considered diagnostic for CeD (21).

Two distinct models have been presented to explain the crypt hyperplasia and increased epithelial turnover in CeD. One model explains the crypt hyperplasia as a mechanism compensatory to increased epithelial cell killing by intraepithelial lymphocytes. Both intraepithelial CD8^+^ T cells carrying αβ T cell receptors (TCRs), and TCR γδ T cells have a cytotoxic potential that involves NK cell receptors recognizing stress-induced molecules expressed by epithelial cells (22–24). These T cells are likely responsible for the increased epithelial cell killing observed in active CeD (25). The alternative model explains crypt hyperplasia as a consequence of increased crypt cell division following activation of T cells. It was demonstrated that activation of lamina propria CD4^+^ T cells leads to crypt hyperplasia (26, 27), and, subsequently, it was shown that cytokines produced by CD4^+^ T cells induce lamina propria fibroblasts to produce keratinocyte growth factor, which acts on crypt cells to instigate increased cell division (28, 29). This model puts the primary driver of crypt hyperplasia at the level of stem cells, yet with cytokines produced by the CD4^+^ T cells acting on these cells indirectly via fibroblasts. The 2 models are not mutually exclusive. An appreciation of the basis for crypt hyperplasia is important not only for understanding the molecular underpinnings of CeD, but also for understanding the cellular programs of the small intestine that are activated by inflammatory cues.

Spatial tissue proteomics is a powerful technology that can elucidate disease mechanisms as recently demonstrated with toxic epidermal necrolysis (30). Here, we have used this technology on the crypt zone of intestinal biopsy sections from patients with CeD who were followed consecutively from having active disease to being in remission on a gluten-free diet. Accompanied by interventional studies in genetically modified mice, the results demonstrate that IFN-γ is a driver of crypt hyperplasia in CeD by acting directly on crypt epithelial cells. Furthermore, the results show that IFN-γ profoundly changes the metabolism of epithelial cells of the crypt zone. These findings have relevance for other inflammatory conditions of the gut in which IFN-γ is a central cytokine.

## Results

### The crypt compartment proteome of the celiac small intestine.

**Figure 1:**
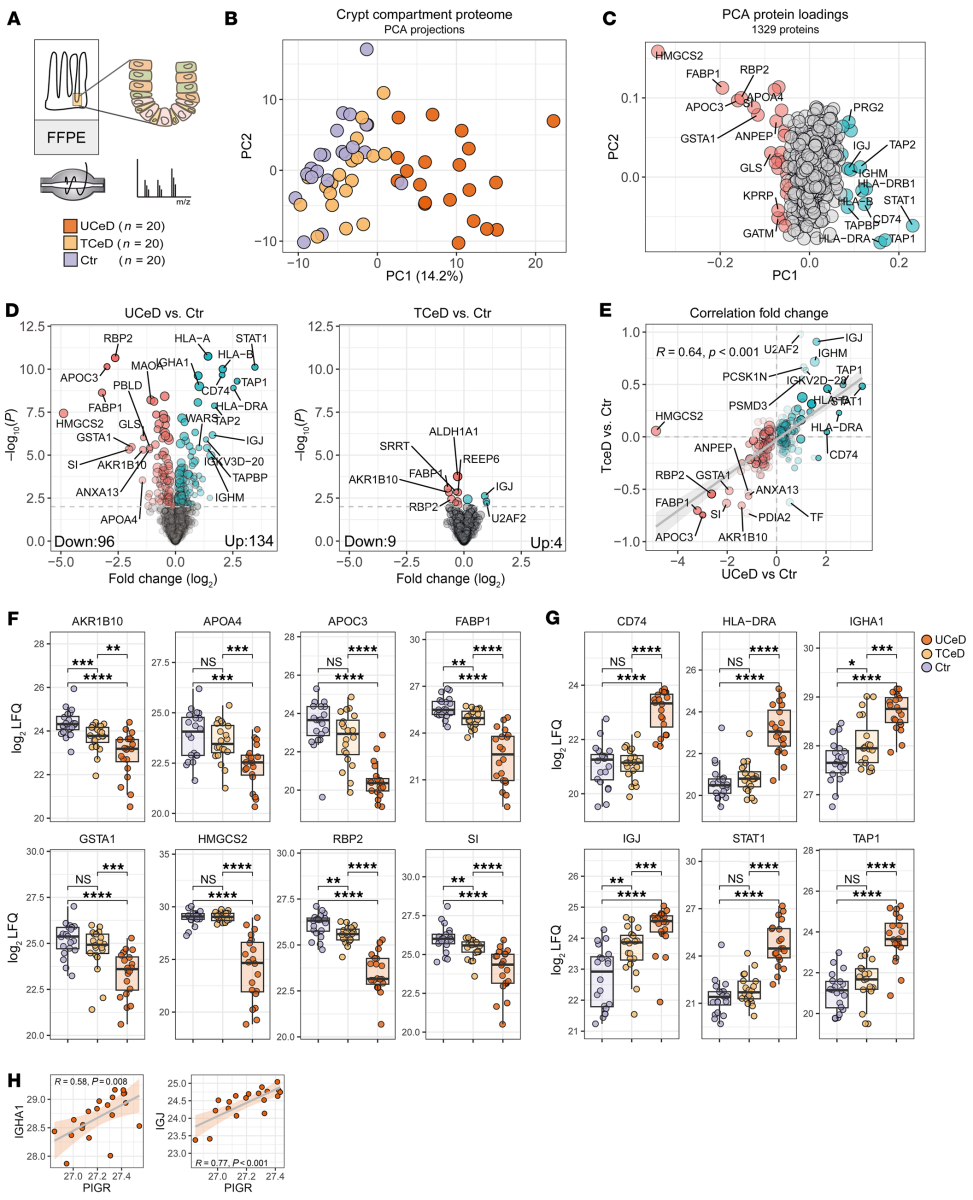
Small intestinal crypt proteome differs in UCeD compared with TCeD and non-CeD controls. (**A**) Schematic of the workflow for spatially resolved proteome analysis of the small intestinal crypt compartment from human FFPE duodenal biopsies. We analyzed biopsies from adults with CeD (*n* = 20) collected at the time of diagnosis (UCeD) and after treatment with a gluten-free diet (TCeD), as well as biopsies from non-CeD individuals (control [Ctr]) (*n* = 20). (**B**) PCA based on the expression of 1,329 proteins. Each data point reflects averaged data per biopsy (*n* = 60). (**C**) Protein loadings that drive separation along PC1 and PC2. (**D**) DEPs in UCeD versus control (left) and TCeD versus control (right). Colored dots indicate significant DEPs comparing UCeD versus control. (**E**) Correlation of log_2_-transformed fold change for UCeD versus control DEPs (*n* = 230). Two-sample Students *t* test; permutation-based FDR control (FDR = 0.05); 250 permutations were applied. *R* = Pearson’s correlation coefficient. (**F** and **G**) log_2_ LFQ protein expression values for selected proteins that drive PC1 and are DEPs. Each dot reflects the mean expression value per biopsy. *P < 0.05, **P < 0.01, ***P < 0.001, and ****P < 0.0001, by Mann-Whitney *U* test with Benjamin-Hochberg correction for multiple testing. (**H**) Correlation of log_2_-transformed LFQ protein expression. Each dot reflects average values for 1 biopsy. *R* = Pearson’s correlation coefficient.

We analyzed archival formalin-fixed, paraffin-embedded (FFPE) biopsies of twenty adult CeD patients both at the time of diagnosis (untreated CeD; UCeD; histology Marsh 3a–c) and in complete histological remission after treatment with gluten-free diet (treated CeD; TCeD; histology Marsh 0). The Marsh scoring was based on routine histology assessments. For comparison, we included from our archival research biobank biopsies 20 non-CeD individuals (controls) with normal gut histology (31) (Supplemental Table 1; supplemental material available online with this article; https://doi.org/10.1172/JCI194858DS1). Tissue sections were stained with hematoxylin and azophloxine to identify Paneth cell granules to ensure the correct identification of well-oriented crypts, followed by laser-capture microdissection (LCM) of regions encompassing Paneth cells, ISCs and newly divided daughter cells (Figure 1A and Supplemental Figure 1). Two replicate samples of pooled crypt regions were collected for all biopsies when possible (*n* = 109) and processed for mass spectrometry–based (MS-based) proteomics analysis for protein identification and label-free quantification (Supplemental Table 2).

### The crypt proteome in UCeD differs from that of TCeD and non-CeD controls.

Principle component analysis (PCA) based on the expression of 1,329 proteins revealed that crypt samples from UCeD biopsies differed from TCeD and control samples (Figure 1B). Key drivers of the separation along PC1 included proteins indicative of inflammatory response, such as STAT1, TAP1/TAP2, CD74, HLA-A, and HLA-DRA, as well as cell metabolism proteins such as HMGCS2, FABP1, RBP2, and AKR1B10 (Figure 1C). These proteins were also among the most significant differentially expressed proteins (DEPs) when comparing UCeD samples with control samples (2-sample Student’s *t* test; total *n* = 230; upregulated in UCeD *n* = 134, downregulated in UCeD *n* = 96) (Supplemental Table 3 and Figure 1D). Only 13 proteins reached statistical significance when comparing TCeD and control samples. However, the majority of proteins that were significantly (*P* < 0.01, Student’s *t* test) changed in UCeD versus control samples (DEP) showed similar directional fold changes when comparing TCeD with control samples (Figure 1E). This suggests varying degrees of low-level gut disease activity in the crypt of TCeD biopsies, which agrees with the positioning of TCeD samples between control and UCeD samples in the PCA plot (Figure 1B). A notable exception was HMGCS2 levels, which were similar in the TCeD and control samples. The key drivers of separation along PC1 were also among the most DEPs. This significance held true when comparing expression in the level of individual proteins and biopsies (Figure 1, F and G). Among the DEPs were IGHA1, which is not expressed by epithelial cells but is transported through epithelial cells via the poly Ig receptor (PIGR). Indeed, both IGHA1 and J-chain (IGJ) signal correlated with PIGR expression (Figure 1H). Collectively, these data show that the crypt compartment proteome in CeD biopsies differed markedly from that of non-CeD controls with differential expression of proteins indicative of inflammation and an altered metabolic profile.

### The proteomic signatures of UCeD crypt compartment reveal a response to IFN-γ and altered fatty acid metabolism.

**Figure 2:**
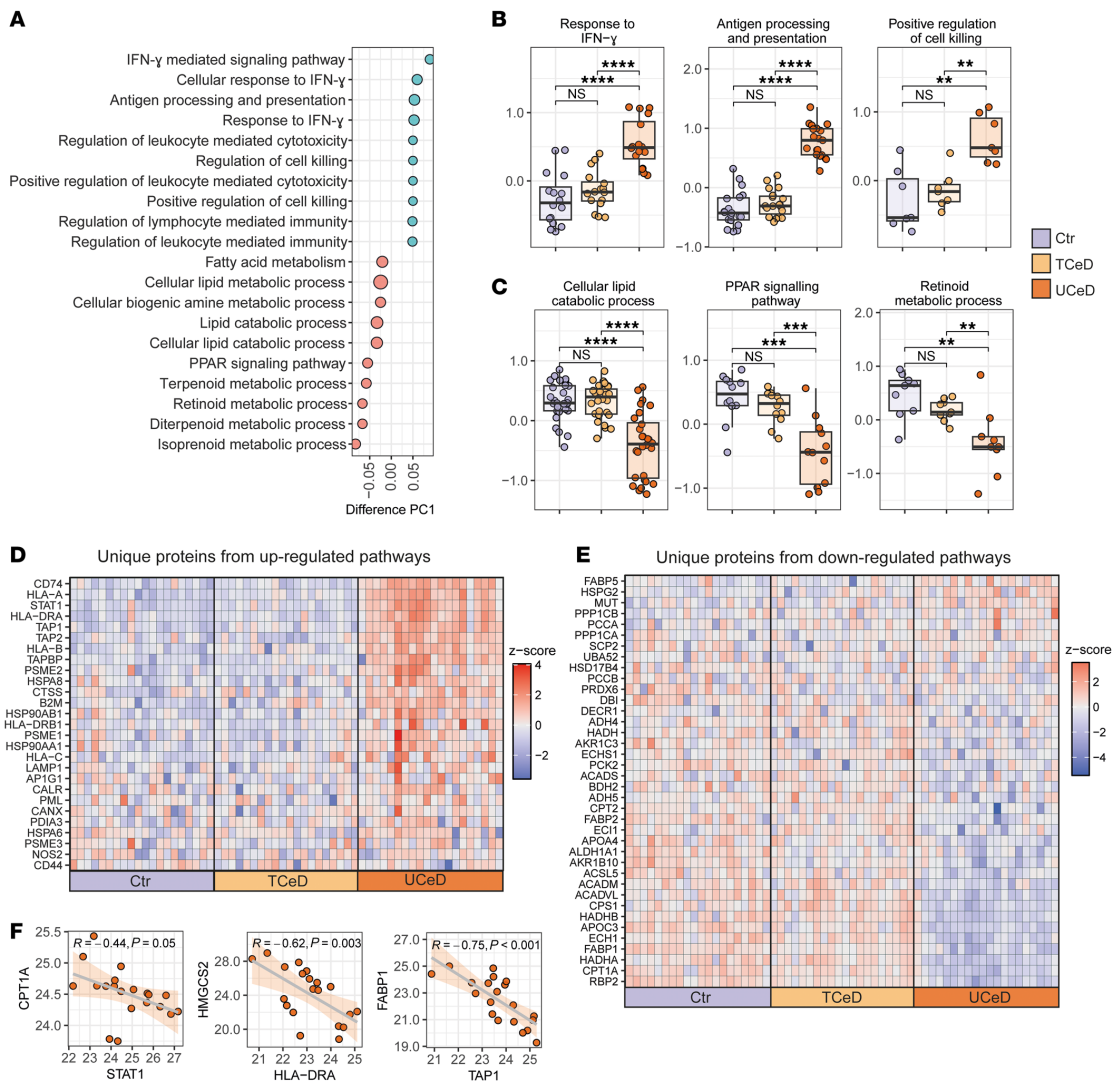
Altered biological pathways in the crypt regions of untreated CeD. (**A**) Top-20 most enriched GO biological processes and KEGG pathways along PC1 from Figure 1C (Benjamin-Hochberg FDR <0.05). (**B** and **C**) *Z*-scored expression of proteins mapped to the indicated enriched pathways. Each data point represents 1 protein and shows the mean *z*-scored expression per group. *P < 0.05, **P < 0.01, ***P < 0.001, and ****P < 0.0001, by Mann-Whitney *U* test with Benjamin-Hochberg correction for multiple testing. (**D** and **E**) Tile plot of *z*-scored protein expression for (**D**) unique proteins (*n* = 27) mapped to upregulated pathways in **B** and (**E**) unique proteins (*n* = 38) mapped to upregulated pathways in **C**. (**F**) Correlation of log_2_-transformed LFQ protein expression. Each data point shows mean values for 1 biopsy.

To determine the biological processes behind the differential proteome expression of UCeD crypts, we performed enrichment analysis of biological pathways along PC1 (Supplemental Table 4). Filtering for Kyoto Encyclopedia of Genes and Genomes (KEGG) pathways and Gene Ontology (GO) biological processes with an adjusted *P* value of less than 0.005 showed enrichment for pathways including “response to IFN-γ” and “antigen processing and presentation” (Figure 2, A and B), which agrees with the increased expression of proteins such as STAT1 and WARS and components of the antigen processing and presentation machinery (TAP1, TAP2, CD74, and HLA molecules) (Figure 1D). We found that pathways reflecting fatty acid metabolism and PPAR signaling were also enriched and were reduced in UCeD crypts (Figure 2, A and C). Comparing individual proteins mapped to the pathways displayed in Figure 2, B and C, confirmed their increased (Figure 2D) and decreased (Figure 2E) expression in UCeD versus TCeD and control crypt samples. Strikingly, the expression of key IFN-γ response proteins in UCeD crypts was inversely correlated with the expression of proteins involved in cell metabolism, as exemplified by correlations of STAT1-CPT1A, HLA-DRA-HMGCS2, and TAP1-FABP1 (Figure 2F). This observation is suggestive of coordinated regulation of these pathways. Moreover, there was a connection in active CeD between the IFN-γ response and tissue remodeling. As part of a previous study, we established histology and proteome scores for the same celiac biopsies (31). Both of these scores serve as proxies for tissue remodeling. We observed strong correlations between the crypt IFN-γ response and the villous height to crypt depth (Vh/Cd) ratio and proteome scores (Supplemental Figure 2). Furthermore, the increased expression of proteins reflecting the response to IFN-γ was not confined to the crypt region but was also observed in the villous epithelial cell layer (Supplemental Figure 3).

### Upregulation of the antigen presentation pathway in UCeD.

**Figure 3:**
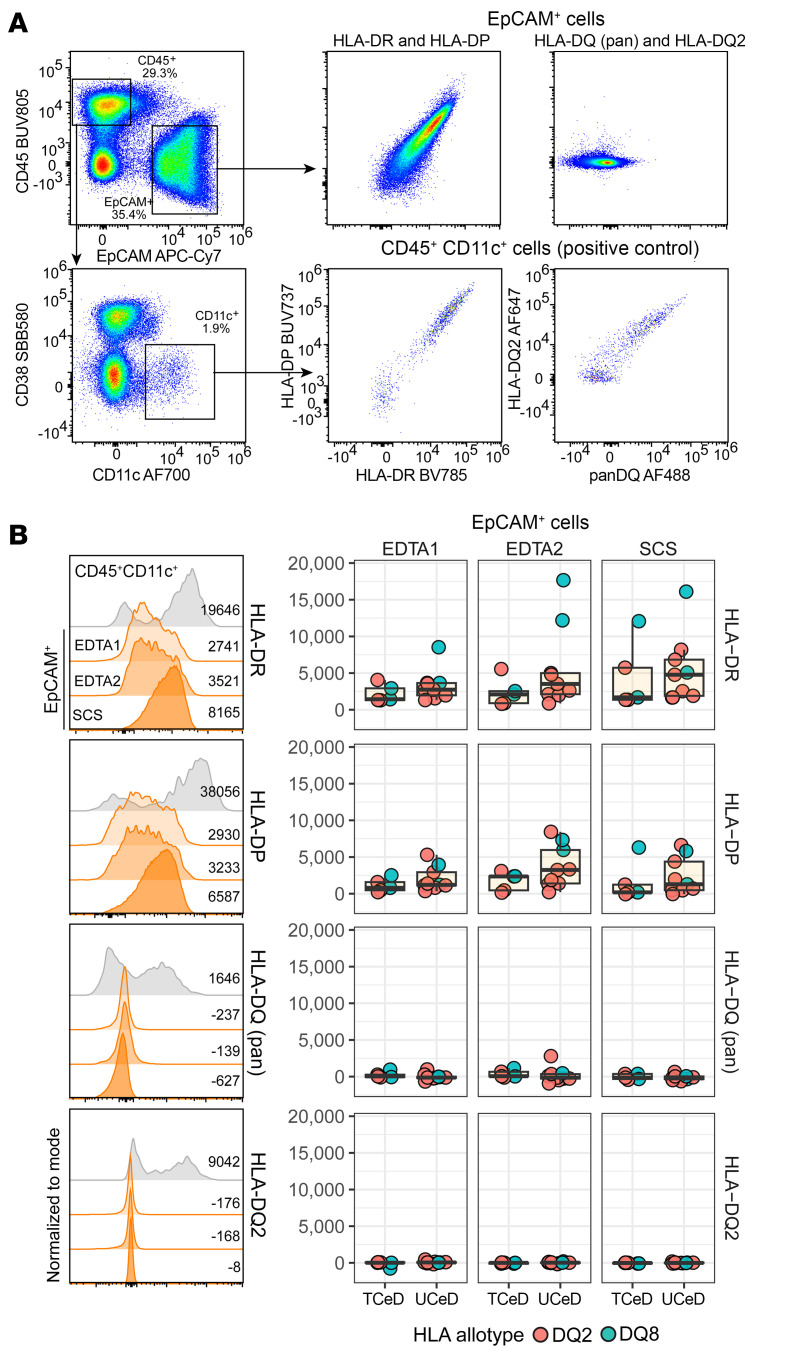
Expression of MHC II molecules on gut epithelial cells in CeD. (**A**) Flow cytometry plots with staining results for the collagenase-treated single-cell suspension (SCS) fraction from an untreated patient with CeD who is HLA-DQ2.5 homozygous. Cells expressing HLA-DQ in this individual should stain both for HLA-DQ (pan) and HLA-DQ2. (**B**) Staining results for HLA-DR, HLA-DP, HLA-DQ (pan), and HLA-DQ2 for 3 cell fractions — EDTA1, EDTA2, and SCS. The left-most column shows histograms for the same individual depicted in **A**, whereas the 3 other columns show staining results as the median fluorescence intensity (MFI) of EpCAM^+^ cells of the 3 cell fractions from 9 patients with UCeD and 5 patients with TCeD.

IFN-γ is a known inducer of antigen-presenting pathway proteins, including MHC class II molecules (32). A study of mice suggested that MHC class II expression on ISCs enables direct interaction of these cells with CD4^+^ T cells, and ISC subsets found to have differential ability to interact with CD4^+^ T cells were defined (33). Relevant to these observations, of 3 defined ISC subsets (ISC-I, ISC-II, and ISC-III) in mice, the crypt proteome data of UCeD were skewed toward the gene set of the ISC-II subset (Supplemental Figure 4A). Moreover, we observed increased expression of HLA-DRA and HLA-DRB1 in crypts of UCeD versus TCeD and controls (Supplemental Figure 4B). To further address the expression of MHC class II molecules on gut epithelial cells, we performed flow cytometric analysis of fresh, noncryopreserved cells prepared from duodenal biopsies from patients with CeD (Supplemental Table 5). Following treatment with EDTA and collagenase, 3 fractions of cells were obtained from 9 patients with UCeD and 5 patients with TCeD (Supplemental Figure 5A). Staining with antibodies specific for HLA-DR, HLA-DP, HLA-DQ, and HLA-DQ2 in EpCAM^+^ cells (Supplemental Figure 5, B–D) revealed cells that stained for HLA-DR and HLA-DP, with higher signal in UCeD than TCeD cells (Figure 3A). No epithelial staining was observed for HLA-DQ in untreated or treated CeD samples, whereas CD11c^+^ cells, as previously demonstrated to be located in the lamina propria (34), stained brightly positive in both conditions (Figure 3B). As could be expected from this result, negative staining for HLA-DQ was also observed in EpCAM^+^CD44^+^CD117^–^CD166^+^CD24^–^ cells (Supplemental Figure 6), which have been demonstrated to encompass ISCs (35).

### Small intestinal remodeling induced by IFN-γ in mice recapitulates key features of CeD.

**Figure 4:**
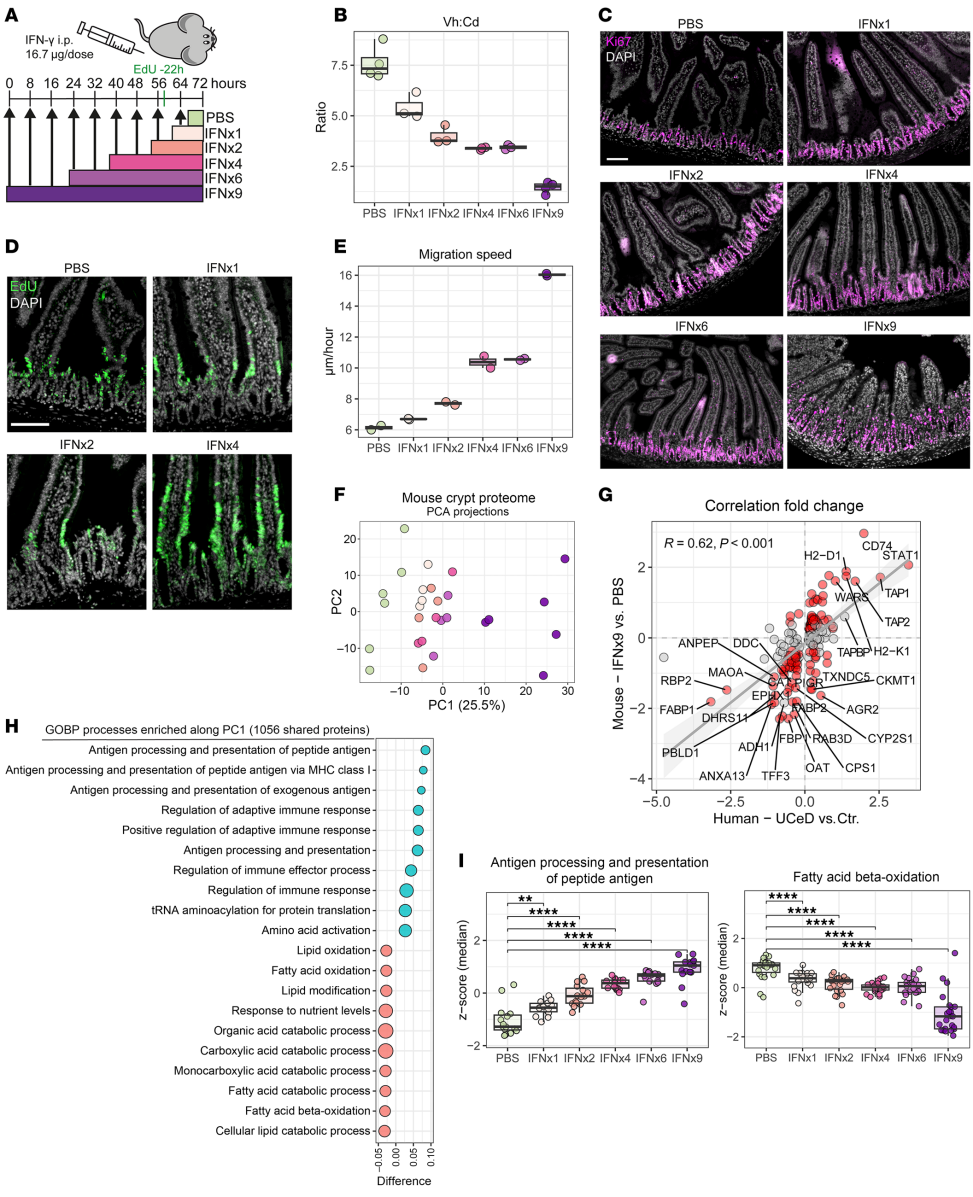
Small intestinal tissue remodeling induced by IFN-γ in mice. (**A**) Scheme depicting the IFN-γ injection regime. Mice were injected intraperitoneally at 8-hour intervals with 16.7 μg IFN-γ in PBS or PBS alone, followed by euthanasia 8 hours after the last injection. A subset of the mice were injected with EdU 22 hours prior to euthanasia. (**B** and **C**) Measurement of the Vh/Cd ratio (**B**) based on Ki67 staining (**C**) to define crypt depth and nuclei staining to define the villous length. Each data point represents 1 mouse, and data were pooled from 3 independent experiments. For each mouse, 3–6 pairs of villous heights and crypt depths were measured per image from 3–5 separate images across the proximal small intestine. (**C**) Representative images of Ki67 staining are shown. Scale bar: 100 μm. (**D** and **E**) Assessment of the cell migration rate from measurement of the EdU front. (**D**) Representative images are shown. Scale bar: 100 μm. For PBS and IFNx4, the same sections are shown in **C** and **D**, with staining for Ki67 and EdU, respectively. (**E**) Migration speed calculated from measurement of the distance from the crypt bottom to the EdU migration front divided by 22 hours. Each data point represents 1 mouse, and data were pooled from 2 independent experiments. For each mouse, 3–5 crypts were measured per image from 5–7 images across the proximal small intestine. The migration speed for IFNx9 reflects an approximation, as EdU^+^ cells had reached the villous tip. (**F**–**I**) Crypt proteome analysis of proximal small intestine from IFN-γ–treated mice. (**F**) PCA based on expression of 3,355 proteins. Each data point represents values averaged per mouse (*n* = 28). (**G**) Correlation of log_2_-transformed fold change for UCeD versus control DEPs and mouse orthologs comparing IFNx9 with PBS. Colored dots reflect DEPs for IFNx9 versus PBS (2-sample Student’s *t* test). (**H**) Top-20 most enriched GO biological processes along PC1 following filtering on proteins that are shared between mouse and human crypt proteome datasets (*n* = 1,056). (**I**) Expression of proteins mapped to the indicated enriched pathways. Each data point represents 1 protein and shows the median *z*-scored expression per treatment group. **P < 0.01 and ****P < 0.0001, by Mann-Whitney *U* test with Benjamin-Hochberg correction for multiple testing.

The pathway enrichment analysis pointed to IFN-γ as the key driver of the crypt proteome changes in UCeD. Because many proteins are shared between cytokine response pathways, we sought to experimentally address whether the observed proteome changes truly reflect IFN-γ–mediated processes. Intraperitoneal injection of IFN-γ into mice induces pronounced remodeling in the small intestine, with crypt hyperplasia and villous shortening (36, 37). To resolve both time and dose dependency of the IFN-γ–mediated intestinal remodeling processes, we injected mice at 8-hour intervals with 1, 2, 4, 6, or 9 doses of 16.7 μg recombinant IFN-γ followed by tissue harvesting 8 hours after the last injection (Figure 4A). A subset of mice were injected with 5-ethynyl-2′-deoxyuridine (EdU) 22 hours prior to tissue harvesting to determine epithelial cell migration speed. Tissue from the proximal one-third of the small intestine was collected, fixed in formalin, and then paraffin embedded for tissue sectioning. We found that IFN-γ injection resulted in a time- and dose-dependent increase in the depth of proliferating crypt cells as determined by staining for Ki67 with an accompanying reduction of the Vh/Cd ratio (Figure 4, B and C). The IFN-γ–induced remodeling also induced a time- and dose-dependent increase in the epithelial cell turnover rate as determined from measurement of the EdU migration front (Figure 4, D and E). We next sectioned FFPE tissue for isolation of crypt regions by LCM and MS-based tissue proteome analysis (*n* = 63). Following filtering, we identified and quantified 3,356 proteins across 52 samples from 28 mice (Supplemental Table 6). PCA revealed a clear dose- and time-dependent effect of IFN-γ on crypt proteome expression (Figure 4F). Among the proteomic signals driving PC1 were enzymes involved in carbohydrate and amino acid metabolism, fatty acid metabolism, as well as Lyz1 (Supplemental Figure 7, A and B). Administration of IFN-γ led to a reduction of Lyz1 (Supplemental Figure 7C), indicating a loss of Paneth cells, which was confirmed by immunofluorescence staining (Supplemental Figure 7D). In UCeD crypts, the proteomic signal for Lyz was reduced but not absent (Supplemental Figure 7E). Comparing the fold change of human DEPs with the fold change of mouse orthologs for mice injected with 9 doses of IFN-γ, we observed clear overlaps in DEPs and similar directional changes in protein expression (Figure 4G). When we filtered for proteins detected in both human and mouse datasets (*n* = 1,056) and analyzed enrichment of GO biological processes along PC1, we found overlap in processes such as antigen processing and presentation and fatty acid metabolism (Supplemental Table 7 and Figure 4H). The expression of proteins mapped to “antigen processing and presentation” and “fatty acid beta-oxidation” changed in an IFN-γ dose- and time-dependent manner (Figure 4I). In summary, the key remodeling events of CeD with crypt hyperplasia — reduced Vh/Cd ratio and increased epithelial cell turnover — also occurred in mice in response to IFN-γ injection. In addition, key biological pathways that were altered in the UCeD crypt compartment, including the altered metabolic state with reduced fatty acid metabolism, were also affected by IFN-γ stimulation.

### Crypt epithelial IFN-γ signaling is a key driver of tissue remodeling and altered crypt metabolic state.

**Figure 5:**
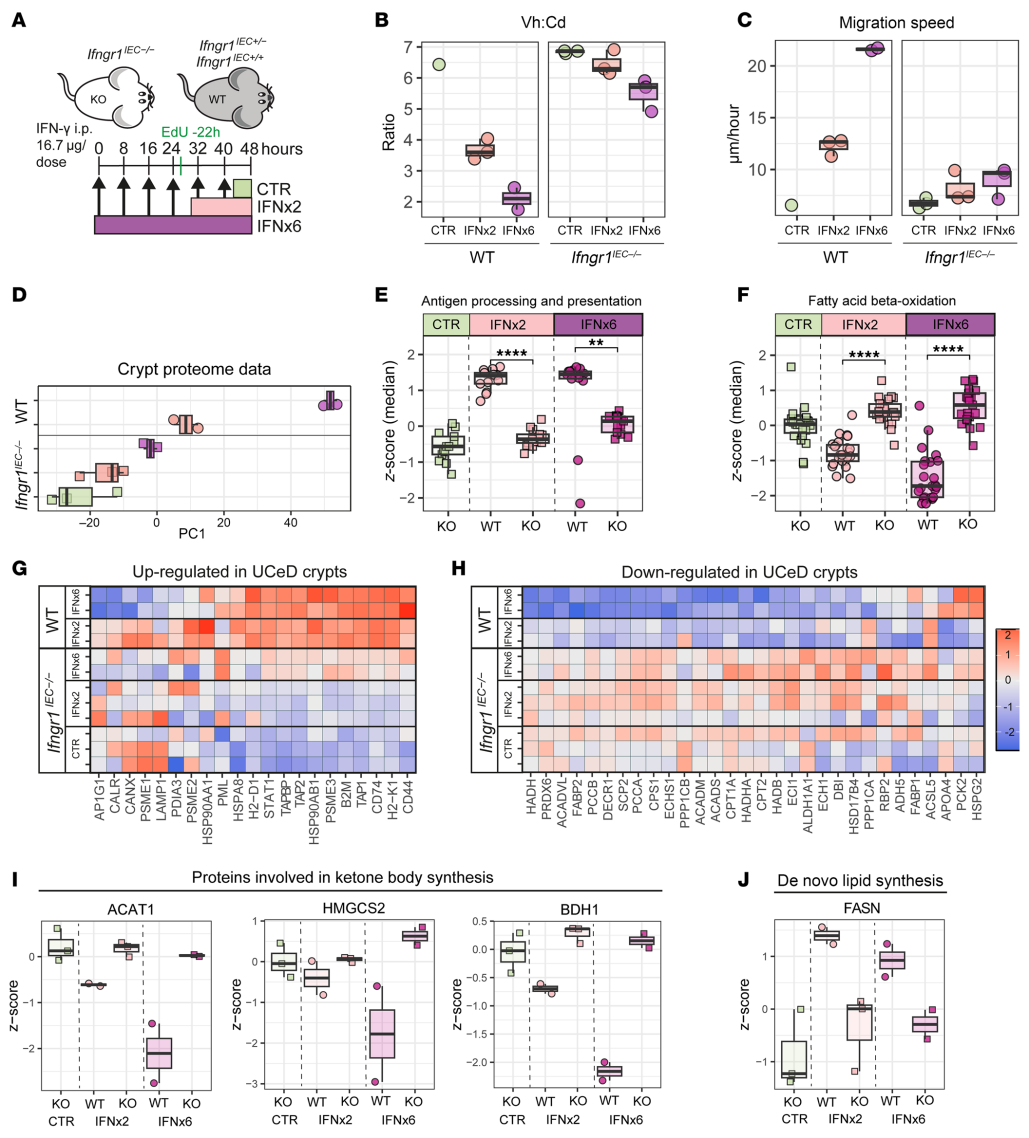
Small intestinal tissue remodeling induced by IFN-γ in mice with targeted deletion of *Ifngr1* in IECs. (**A**) Schematic depiction of the IFN-γ injection regime for mice with deletion of *Ifngr1* (*Ifngr1^IEC−/−^*; KO) in IECs and the corresponding littermate controls (WT). (**B** and **C**) Measurement of the Vh/Cd ratio and epithelial cell migration speed based on EdU staining. For IFNx6 WT mice, the EdU migration front had reached the villi tips. (**D**) Crypt proteome analysis revealed the separation of samples along PC1 according to *Ifngr1* status and IFN-γ injection. Each data point reflects 1 mouse. (**E** and **F**) Expression of proteins mapped to the indicated pathways. Each data point represents 1 protein and shows the median *z*-scored expression level per group. **P < 0.01 and ****P < 0.0001, by Mann-Whitney *U* test with Benjamin-Hochberg correction for multiple testing. (**G** and **H**) Tile plots of *z*-scored expression of proteins from pathways (**G**) upregulated in UCeD (Figure 2D; *n* = 21 mouse orthologs) and (**H**) downregulated in UCeD (Figure 2E; *n* = 30 mouse orthologs). (**I** and **J**) Expression (*z*-scored) of selected proteins involved in ketone body synthesis (**I**) and FASN involved in de novo lipid synthesis (**J**). Each data point reflects 1 mouse.

As the IFN-γ receptor is expressed on many cell types, we next sought to determine whether IFN-γ–induced remodeling processes reflect indirect effects or direct effects from IFN-γ signaling in the intestinal epithelial cell (IEC) compartment. To this end, we generated IEC-specific IFN-γ receptor 1–KO mice (*Ifngr1^IEC−/−^* mice; *Ifngr1^fl/fl^* *Villin*-Cre mice), which, together with littermate controls (WT), were injected intraperitoneally with 2 or 6 doses of IFN-γ as well as EdU 22 hours prior to euthanasia and analysis (Figure 5A). We found that targeted deletion of *Ifngr1* in the epithelial compartment prevented IFN-γ–induced tissue remodeling with a reduction in the Vh/Cd ratio (Figure 5B). The effect was evident in the crypt compartment (Supplemental Figure 8A), as *Ifngr1^IEC−/−^* mice presented with no increase in crypt depth, yet we observed a small, dose-dependent IFN-γ–induced decrease in villous height (Supplemental Figure 8B). The targeted *Ifngr1* deletion also prevented IFN-γ–induced crypt hyperproliferation, as determined by EdU incorporation (Figure 5C). Proteome analysis of LCM isolated crypt regions (Supplemental Table 8) revealed a clear separation along PC1 for samples from WT mice injected with IFN-γ, which was in contrast to samples from *Ifngr1^IEC−/−^* mice that received no treatment or that were treated with IFN-γ (Figure 5D). Indeed, proteins mapped to “antigen processing and presentation” and “fatty acid beta-oxidation” were altered in IFN-γ–treated WT mice (Figure 4I and Figure 5, E and F), but not in IFN-γ–treated *Ifngr1^IEC−/−^* mice (Figure 5, E and F). We found that the expression of mouse homologs to proteins from up- or downregulated biological pathways in UCeD crypts (Figure 2, D and E) increased and decreased, respectively, upon IFN-γ treatment in WT mice, but not in *Ifngr1^IEC−/−^* mice (Figure 5, G and H). Similarly, we observed upon IFN-γ treatment a reduced expression of key enzymes involved in the synthesis of ketone bodies (ACAT1, HMGCS2, and BDH1) in WT mice but not in *Ifngr1^IEC−/−^* mice (Figure 5I). Interestingly, the IFN-γ–induced decrease in lipid metabolism was paralleled by an increase in FASN expression, which was indicative of increased de novo lipid synthesis (Figure 5J). Protein-protein interaction analysis indicated that proteins from up- or downregulated biological pathways in UCeD crypts (Figure 2, D and E) form functional networks that reflect intestinal remodeling mediated by IFN-γ through IFN-γ receptor and STAT1 signaling (Supplemental Figure 9).

### Different kinetics of IFN-γ–driven alterations in proteomic signatures.

**Figure 6:**
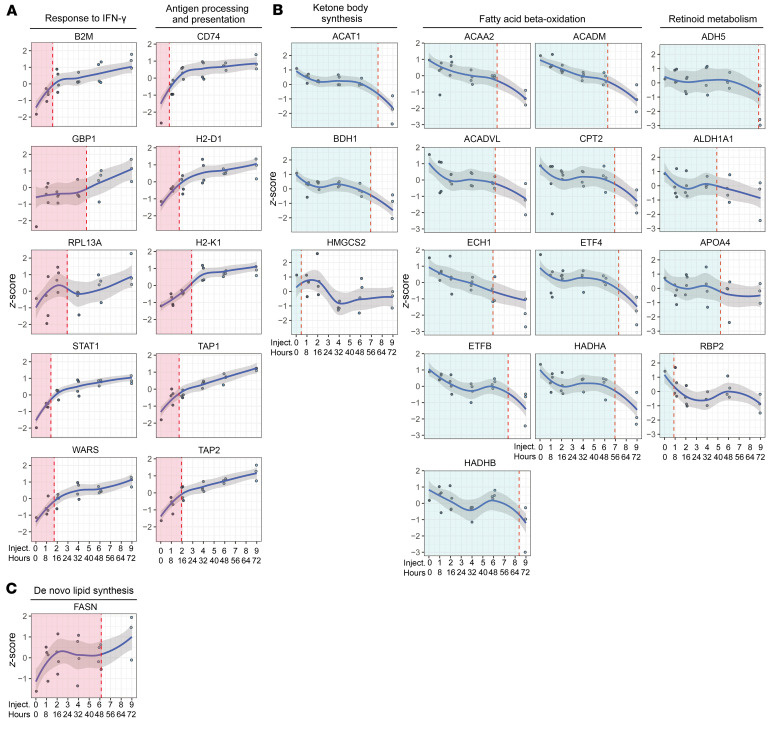
Kinetics of IFN-γ–driven alterations in proteomic signatures of crypt region cells of mice. Time- and IFN-γ dose–dependent increase (**A** and **C**) or decrease (**B**) in mouse crypt expression of proteins that represent selected GO biological processes that are altered in UCeD crypts. Each data point reflects 1 mouse. Smoothed curves were generated using LOESS regression (blue line). Shaded regions represent 95% CIs for the fitted curve. Vertical line (red) indicates the model point at 50% change in protein expression. Colors indicate proteins that increased (light red) or decreased (light blue) in response to IFN-γ.

To gain better insight into the link between the crypt response to IFN-γ and the altered cell metabolic profile in the crypt zone, we analyzed proteomic changes in WT mice over the time course (8–72 hours) of 1–9 injections of IFN-γ. One injection of IFN-γ into WT mice was sufficient to induce crypt expression of direct IFN-γ response proteins such as STAT11, WARS, and B2M (Figure 6A). Similar kinetics were observed for proteins involved in the antigen presentation machinery (CD74, TAP1, TAP2, H2-D1, H2-K1) (Figure 6A). Proteins reflecting ketone body synthesis, fatty acid β-oxidation, and retinoid metabolism (Figure 6B) decreased in a dose- and time-dependent manner, but generally with more protracted kinetics. FASN, indicative of de novo lipid synthesis, increased already following 1 injection of IFN-γ (Figure 6C). Overall, these data point to a coordinated response to IFN-γ involving both antigen presentation machinery and cell metabolic changes.

## Discussion

Using spatial tissue proteomics, we demonstrated that IFN-γ is a driver of crypt hyperplasia in CeD by acting directly on crypt epithelial cells. The changes were strikingly paralleled by changes in cell metabolism, which suggests that epithelial cell proliferation and hyperplasia are linked with metabolic changes. The implications of the findings go beyond CeD, as crypt hyperplasia is observed in many inflammatory conditions of the small intestine, like tropical sprue, bacterial overgrowth syndrome, autoimmune enteropathy, and graft-versus-host disease (2).

Our findings place T cells via the production of IFN-γ as the driver of crypt hyperplasia by action on epithelial cells in the crypt region. The T cells involved could be CD4^+^ T cells in the lamina propria or intraepithelial CD8^+^ T cells, as both cell types produce IFN-γ. In the active CeD lesion, intraepithelial CD8^+^ T cells seem to produce more IFN-γ than do lamina propria CD4^+^ T cells (16, 17). Notwithstanding, as the gluten-specific CD4^+^ T cells in CeD are located in the lamina propria spatially close to the crypts (11, 38), and intraepithelial CD8^+^ cells typically accumulate at the villous tip distant to the crypt zone (39), the gluten-specific CD4^+^ T cells may be particularly important for mediating IFN-γ effects on crypt epithelial cells in CeD.

The IFN-γ–induced signature in crypt epithelial cells includes a program for “antigen presentation” with upregulated expression of invariant chain (CD74) and MHC class II molecules. This signature points to a role of epithelial cells in antigen presentation to CD4^+^ T cells. In fact, in mouse models, it was demonstrated that ISCs are involved in antigen presentation to CD4^+^ T cells (33) and, further, that IECs in organoid cultures can present gluten antigen (40). Staining of fresh epithelial cells prepared from duodenal biopsies from patients with CeD confirmed the epithelial cell expression of MHC class II molecules (both HLA-DR and HLA-DP), and we observed increased expression in the cells of untreated patients compared with those of patients in remission. This finding indeed suggests an antigen-presenting role of epithelial cells for CD4^+^ T cells. We were particularly interested in addressing whether epithelial cells, and especially ISCs, can be involved as antigen-presenting cells in the IFN-γ–controlled circuit that gives crypt hyperplasia in CeD. Importantly, only disease-associated HLA-DQ allotypes are involved in presentation of gluten peptides to CD4^+^ T cells in CeD (10). In contrast to a prior study (40), but in accordance with several other studies (38, 41–43), we observed no expression of HLA-DQ by epithelial (EpCAM^+^) cells, including CD44^+^CD117^–^CD166^+^CD24^–^ cells that should encompass ISCs (35). Epithelial cells in culture on stimulation by IFN-γ show differential expression of MHC II isotypes, with a higher concentration of IFN-γ needed for the expression of HLA-DQ (44, 45). This observation likely explains the absence of duodenal epithelial HLA-DQ expression in CeD. Given these findings, we conclude that, within the gut mucosa of the CeD lesion, there are other antigen-presenting cells than epithelial cells, like HLA-DQ expressing CD11c^+^ DCs (34) or possibly plasma cells (46), that present antigen to gluten-specific CD4^+^ T cells.

Guy-Grand et al. demonstrated that systemic administration of IFN-γ to mice leads to crypt cell hyperplasia, villous shortening, and increased crypt area suggestive of increased cell divisions (36). These changes did not require the presence of intraepithelial lymphocytes, as the changes were also observed in mouse strains lacking intraepithelial lymphocytes, including germ-free mice, common cytokine receptor γ-chain–mutant mice, and RAG-2/γ chain double-mutant mice. Interestingly, the fact that intraepithelial lymphocytes were not required for the effects of IFN-γ contrasted with the changes induced by IL-12. While systemic administration of IL-12 led to increased epithelial cell turnover and also villous epithelial cell damage, these effects necessitated the presence of intraepithelial lymphocytes. Eriguchi et al., on administering IFN-γ to mice, also observed small intestinal crypt hyperplasia and villous shortening, as well as changes in ISC phenotypic markers (37). Takashima and coworkers, on studying intestinal graft-versus-host disease, a condition marked by crypt cell hyperplasia with increased proliferating Ki67^+^ cells, observed increased death of ISCs being mediated by IFN-γ (47). They later reported that these graft-versus-host manifestations, including crypt cell proliferation, involve STAT1 and the IFN-γ receptor of gut epithelial cells and, further, that low doses of IFN-γ induce cell divisions in epithelial-only organoid cultures (48). Omrani et al., studying age-related changes in the gut, concluded that by older age there is increased production of IFN-γ by T cells in the lamina propria and that IFN-γ acting on crypt cells gives ISC activation and exit from a stem state toward cells that express MHC II genes that are proliferating and transcriptionally primed toward the secretory lineage (49). In addition, the authors observed a reduction of ISCs in older mice. In gut biopsies from patients with CeD in remission, who had been orally challenged with gluten, there is an increased goblet cell signature (50, 51) — observations which are in line with a disease-related bias of ISCs toward secretory fate. Studies addressing the effect of IFN-γ on Paneth cells have reported that IFN-γ induces apoptosis of these cells (37, 52, 53), and also that IFN-γ is a trigger for Paneth cell degranulation and extrusion (53). We also observed effects of IFN-γ on Paneth cells in mice with reduced lysosome expression on prolonged injection of the cytokine. Studies of CeD have reported diminished or normal numbers of Paneth cells in the active lesion (54–56), less lysozyme activity in CeD jejunal tissue (54), and also that the cells have reduced lysozyme content (55). Our proteomics data are in agreement with these findings, as we observed lower lysozyme protein expression in crypt regions of patients with active CeD compared with controls. Thus, in essence, IFN-γ induces a loss of ISCs, but at the same time the cytokine instigates a program that results in increased cell proliferation in the transit amplifying zone, thereby giving crypt hyperplasia and increased epithelial cell turnover. Taken together, the findings of the literature align well with our notion that crypt hyperplasia in CeD is mainly caused by a direct effect of IFN-γ on crypt epithelial cells.

In mice, dietary interventions have been demonstrated to affect the expression of HMGCS2 and CPT1A in ISCs (57, 58). A high-glucose diet was shown to reduce the expression of HMGCS2 (57), similar to what we observed in the crypt zones of untreated CeD and of mice treated with IFN-γ. Gebert et al., analyzing proteomic changes in intestinal crypts on aging in mice, observed both increased expression of MHC class II molecules and CD74 and, interestingly, reduced expression of HMGCS2 (59). This observation suggests that the induced IFN-γ immune signature and the metabolic changes with reduced expression of key metabolic enzymes are causally related. Furthermore, similar to what has been reported for IFN-γ–treated mice, impaired function and a reduced number of ISCs were observed in mice with genetic ablation of *Hmgcs2* in Lgr5^+^ ISCs (57). Given this scenario, it seems likely that the damage of ISCs being induced by IFN-γ is mediated by the changes in crypt cell metabolism. Changes in crypt cell metabolism could also be involved in the IFN-γ–induced bias toward the secretory linage, as well as the increased proliferation of cells causing crypt hyperplasia. Relevant to these considerations is the observation that a high-fat diet in mice leads to decreased epithelial cell expression of MHC class II molecules, which was mechanistically proven to be mediated by diminished production of IFN-γ due to a reduction of intestinal microbiome diversity (60). It remains a possibility that the effects of a high-glucose diet on the crypt cell metabolism could be mediated at least in part by an immune reaction to an altered microbiota with more production of IFN-γ.

While our results align with the model proposed by MacDonald et al. (28, 29) in that the primary driver of crypt hyperplasia is at the level of ISCs, it differs from this model in that the effect of T cells is mediated directly by IFN-γ on and not indirectly via lamina propria fibroblasts by production of keratinocyte growth factor. The results should, however, not be taken to imply that lamina propria fibroblasts, which consist of several subtypes (61), are not involved in the pathogenesis of CeD. There are several candidate pathways for involvement. Intestinal fibroblasts are critical for the formation of the ISC niche by providing structural support and producing several factors of importance for ISC differentiation (62), as well as producing IL-6, which is important for plasma cell survival (63). As recently demonstrated using an organoid model, fibroblasts also produce the B cell and T cell growth factor IL-7 after stimulation by activated gluten-specific CD4^+^ T cells (64).

Our findings indicate that increased cell division in the transit-amplifying zone, resulting in crypt hyperplasia and increased cell turnover, is part of an active program instigated at the stem cell zone, rather than solely a mechanism to compensate for loss of epithelial cells killed toward the villous tip. The results give support to a model of crypt hyperplasia as a consequence of increased crypt cell division following T cell activation, yet the effect is caused by a direct action of IFN-γ on crypt epithelial cells, not an indirect action involving stromal fibroblasts. As crypt hyperplasia, increased epithelial cell turnover, and villous blunting are observed in many inflammatory conditions of the small intestine, these changes are probably integral parts of a defense program operating in conditions in which IFN-γ is the central inflammatory mediator.

## Methods

### Sex as a biological variant

Duodenal biopsies from both female and male adult patients were used for spatial proteomics analysis and flow cytometry analysis. Experiments on mice with repeat injection of IFN-γ were done using female C57BL/6 mice. Experiments on mice with targeted deletion of *Ifngr1* were performed on both female and male littermates. The findings are expected to be relevant to both males and females, although no experiments were performed to test for sex-related differences.

### Human participants

For spatial proteomics analysis, we used archival FFPE duodenal biopsy material from adults who underwent gastroduodenoscopy at Oslo University Hospital-Rikshospitalet between 2012 and 2023. The patient cohort has previously been described (31), in addition to 4 individuals who were not included in the previous study (2 CeD patients and 2 controls). In total, we analyzed FFPE tissue sections from 20 adults with biopsy-proven CeD, comparing biopsies collected at the time of diagnosis (UCeD, Marsh 3) with biopsies collected at follow-up after treatment with a gluten-free diet (TCeD, Marsh 0). As disease controls, we analyzed biopsies from 20 non-CeD adults with normal duodenal histology (Marsh 0) (Supplemental Table 1). For flow cytometric analysis of gut epithelial cell MHC II expression, we analyzed biopsies from 9 patients with UCeD and 5 patients with TCeD who visited the endoscopy unit at Oslo University Hospital–Rikshospitalet for diagnostic examination and clinical follow-up of CeD (Supplemental Table 5). The diagnosis of CeD was made according to established guidelines (21).

### Mouse experiments

C57BL/6J mice (9- to 11-week-old females; Janvier Lab) were injected intraperitoneally with recombinant IFN-γ (GenScript, Z02916-1) dissolved in sterile Dulbecco’s PBS. Single doses of 16.7 μg IFN-γ in 200 μL sterile PBS were injected at 8-hour intervals followed by euthanasia 8 hours after the last injection. Four independent experiments were performed on 28 mice that were either treated with PBS (*n* = 6) or IFN-γ in 1 dose (*n* = 4), 2 doses (*n* = 4), 4 doses (*n* = 4), 6 doses (*n* = 4), or 9 doses (*n* = 6). In 2 of the experiments, mice were also injected w intraperitoneally with 100 μL of 0.1 mg EdU in 10% DMSO/PBS) 22 hours prior to euthanasia.

To generate mice with targeted deletion of *Ifngr1* in the epithelial cell compartment, *Ifngr1^fl/fl^* mice (stock no. 025394, The Jackson Laboratory) (65) were crossed with heterozygous Villin-Cre (Vil1-Cre) transgenic mice (stock no. 021504, The Jackson Laboratory) (66), both on a C57BL/6 background. From the filial 1 (F1) progeny, *Ifngr1^fl/WT^* Vil1-Cre–transgenic mice were bred with *Ifngr1^fl/fl^* mice to generate *Ifngr1^fl/fl^* Vil1-Cre–transgenic *Ifngr1^IEC–/–^* mice. Vil1-Cre^–^ littermates with WT expression of *Ifngr1* in epithelial cells were used as control (WT) mice for the IFN-γ injection experiments. Mice (9–11 weeks of age) with targeted deletion of *Ifngr1* (*n* = 9) or Vil1-Cre^–^ littermate controls (*n* = 6) were untreated (*n* = 4) or treated with 2 doses (*n* = 6) or 6 doses (*n* = 5) of IFN-γ as well as with EdU injection 22 hours prior to euthanasia.

Mice were euthanized by cervical dislocation 8 hours after the last injection with IFN-γ. The small intestine was quickly harvested and flushed with ice-cold PBS. From all mice, the proximal one-third of the small intestine was collected and fixed in neutral buffered formalin in the dark overnight at room temperature. From 4 mice (PBS *n* = 2; IFN9 *n* = 2), the middle two-thirds and distal third of the small intestine were also collected. After fixation, 4–7 pieces of approximately 4–5 mm in size were cut tangentially and oriented in Tissue-Tek 2-lane paraform gels (Sakura) for paraffin embedment.

### Tissue sectioning and LCM

#### Human samples.

For human duodenal biopsies, 8 μm FFPE tissue sections were adhered to UV-treated, PEN-covered slides (Zeiss). Sections were dewaxed in xylene (twice) hydrated in 100% ethanol, 95% ethanol, and 70% ethanol (1 minute each), followed by twice for 1 minute in water. Sections were stained with Mayer’s hematoxylin solution for approximately 30 seconds, rinsed in tap water, and incubated in hexamine (1 minute), followed by 1 minute in water, 30 seconds in Azophloxine solution, and then rinsing in water and airdrying. For mouse intestinal sections, 5 μm FFPE tissue sections were adhered to UV-treated, PEN-covered slides (Zeiss) followed by staining with hematoxylin as previously described (50). Sections were stored in dehydrator boxes and dried at 37°C prior to LCM. Crypt regions were isolated using a PALM MicroBeam LCM system (Carl Zeiss MicroImaging), with tissue captured into 0.5 mL opaque adhesive-cap tubes (Zeiss). Crypt regions that encompassed ISCs, Paneth cells, as well as 4–5 daughter cells were isolated. In sections from human biopsies, well-oriented crypts were identified on the basis of a visual presence of Azophloxine-stained Paneth cells. Two replicate samples were collected for most biopsies (~150 000 μm^2^ per sample; *n* = 109).

#### Mouse samples.

Tissue sections (5 μm) from FFPE mouse intestine were adhered to UV-treated, PEN-covered slides (Zeiss), dewaxed as described above, and stained with Mayer’s hematoxylin solution. Crypt regions were isolated using a PALM MicroBeam LCM system (Carl Zeiss MicroImaging), capturing tissue into 0.5 mL opaque adhesive-cap tubes (Zeiss). Crypt regions that encompassed ISCs, Paneth cells, as well as 4–5 daughter cells were isolated according to visual assessment of tissue orientation in intestinal cross-sections. Crypt regions were isolated from the proximal part of the small intestine unless otherwise specified. For each sample, crypts were isolated and pooled from all embedded tissue pieces, with 1–3 replicate samples collected per tissue block (*n* = 63 samples from 28 mice). From 4 mice, crypts from the middle and distal intestine were also collected and processed for MS analysis.

### Sample processing, LC-MS/MS, and raw data analysis

LCM-isolated samples were processed as previously described (50). Dissected tissue was retrieved from adhesive caps using ammonium bicarbonate (10 μL, 50 mM) with ProteaseMax Surfactant (0.2%) (Promega) followed by ammonium bicarbonate (10 μL, 50 mM) and then transferred into 0.5 mL LoBind tubes (Eppendorf). Samples were heated to 98°C for 90 minutes followed by sonication in a water bath for 60 minutes. Disulfide bridges were reduced by addition of dithiotreitol (2 μL, 0.1 M) followed by incubation for 20 minutes at 56°C and then alkylated by addition of iodoacetamide (2 μL, 55 mM) followed by incubation for 15 minutes in the dark at room temperature. Samples were digested by addition of trypsin (1.5 μL, 10 μg/g) and incubated overnight at 37°C. Peptides were purified by solid-phase extraction either by using stage tips with 3 layers of C18 Empore Extraction Disks of C18 micro columns, or loaded onto EvoTips according to the manufacturer’s instruction.

Crypt samples (*n* = 109) from human biopsies were analyzed using an EVOSEP liquid chromatography system connected to a quadrupole – Orbitrap (QExactive HF) mass spectrometer (ThermoElectron) equipped with a nanoelectrospray ion source (EasySpray, Thermo Fisher Scientific). For liquid chromatography (LC) separation, a 15 cm C18 column (column details: 15 μm beads, 150 μm inner diameter, 15 cm long, EV-1074) was used. The standard Evosep (Evosep Biosystems) method of 30 samples/day was used. The mass spectrometer was operated in the data-dependent mode to automatically switch between MS and tandem MS (MS/MS) acquisition. Survey full-scan MS spectra (from *m/z* 375 to 1,500) were acquired in the Orbitrap with a resolution of *R* = 60,000 at *m/z* 200 (after accumulation to a target of 3,000,000 ions in the quadruple). The method used allowed sequential isolation of the most intense multiple-charged ions, up to 7, depending on signal intensity, for fragmentation on the HCD cell using high-energy collision dissociation at a target value of 100,000 charges or a maximum acquisition time of 110 ms. MS/MS scans were collected at 60,000 resolution in the Orbitrap cell. Target ions already selected for MS/MS were dynamically excluded for 30 seconds. General MS conditions were as follows: electrospray voltage, 2.0 kV; no sheath and auxiliary gas flow; a heated capillary temperature of 250°C; and a normalized HCD collision energy of 28%.

Crypt samples from mice injected with IFN-γ and control mice (*n* = 63) were processed and analyzed by ultra-high-performance LC (UHPLC) and trapped ion mobility spectrometry time-of-flight (TIMS-TOF) MS. Digested peptides were cleaned by solid-phase extraction using stage tips with 3 layers of C18 Empore Extraction Disks, vacuum dried, and redissolved in 0.1% formic acid (FA) before injection into a nanoElute UHPLC coupled to a timsTOF fleX or a timsTOF Pro2 mass spectrometer via a CaptiveSpray ion source (all from Bruker Daltonics). For LC separation, a 25cm C18 column (Column details: 1.6 μm beads, 120 Å pore size, 75 μm inner diameter, 25 cm long, Aurora UHPLC column, IonOptics) was used with a flow rate of 0.3 μL/min. Solvent A (0.1% FA) and solvent B (acetonitrile in 0.1% FA) were used for a gradient of 0% to 35% solvent B in 60 minutes. Crypt samples from C57Bl/6J mice injected with IFN-γ were run as 4 independent experiments on a timsTOF fleX operated in data-dependent acquisition (DDA) parallel accumulation serial fragmentation (PASEF) mode, recording mass spectra for MS and tandem MS (MS/MS) scans between 100 and 1,700 *m/z*. The ion mobility resolution was 0.60 to 1.60 V × s/cm^2^ over a 100 ms ramp time over a 100 ms ramp time. Ten PASEF MS/MS scans per cycle with a nearly 100% duty cycle were used for data-dependent acquisition. We applied a polygon filter in the *m/z* and ion mobility space to exclude low *m/z* singly charged ions from the PASEF precursor selection and applied an active exclusion time of 0.4 minutes to precursors that reached 20,000 intensity units. Collisional energy was ramped stepwise as a function of ion mobility.

Crypt samples from *Ifngr1^IEC–/–^* mice and littermate controls (*n* = 15) were analyzed on a timsTOF Pro2 instrument operating in data-independent acquisition (DIA) PASEF mode. Mass spectra for MS were recorded between *m/z* 100 and 1,700. Ion mobility resolution was set to 0.85–1.30 V × s/cm^2^ over a ramp time of 100 ms. The MS/MS mass range was limited to *m/z* 475–1,000 and the ion mobility resolution to 0.85–1.30 V × s/cm^2^ to exclude singly charged ions. The estimated cycle time was 0.95 seconds with 8 cycles using DIA windows of 25 Da. Collisional energy was ramped from 20 eV at V × s/cm^2^ to 59eV at 1.60 V × s/cm^2^.

### Protein identification and data processing

#### Data from human samples.

MS raw files from human crypt samples (.raw files; *n* = 109) were processed in the MaxQuant environment (version 1.6.1.0, Max-Planck-Institute of Biochemistry) (67) with the integrated Andromeda search engine for peptide and protein identification and an FDR threshold of 0.01 for peptide and protein identification. The human UniProtKB FASTA database (September 2018; 20,394 entries) was used as a forward database for protein identification. Match between runs was enabled, and label-free protein quantification (LFQ) was performed using the MaxQuant’s Label Free Quantification algorithm with a minimum ratio count of one. Methionine oxidation and N-terminal acetylation were used as variable modifications and carbamidomethyl cysteine as fixed modification. Processing of MS raw files from LCM-isolated villous epithelium (*n* = 16 samples isolated from *n* = 3 UCeD, *n* = 2 TCeD, and *n* = 2 control biopsies) has previously been described (68).

The ProteinGroups.txt output was processed in Perseus (version 1.6.15.0) (69). For all datasets, proteins matched to the reverse decoy database, identified by site or as potential contaminants, were removed. LFQ expression data were log_2_ transformed. Data were filtered to keep only proteins with valid LFQ values in at least 70% of the samples in at least 1 of the 3 sample groups (UCeD, TCeD, or control), resulting in 1,333 protein groups, of which 1,329 were annotated to a gene name. Missing values were imputed for each sample on the basis of normal distribution to simulate low-abundance LFQ values, and replicate samples from the same biopsy tissue block were averaged prior to statistical analysis. Data were *z*-scored for each row using the median value. Biological pathways were annotated to proteins in Perseus, selecting GO biological processes, GO molecular function, GO cellular component, and KEGG.

#### Data from mouse samples.

MS raw files (.d) files from mouse crypt samples (*n* = 63) analyzed by DDA-PASEF MS were processed with MSFragger (version 4.1) (70, 71) via FragPipe (version 22.0) using the mouse UniProtKB FASTA database (January 2019; 17,006 entries) as a forward database. Methionine oxidation and N-terminal acetylation were used as variable modifications and carbamidomethyl cysteine as fixed modification. Proteins were quantified by MaxLFQ using IonQuant. MS raw files (.d) files from mouse crypt samples analyzed by DIA-PASEF MS (*n* = 15) were processed with MSFragger (version 4.1) calling DiaNN via FragPipe (version 22.0) using the default parameters of the DIA SpecLib_Quant_diaPASEF workflow with MaxLFQ quantification of proteins.

The report.pg.matrix.tsv outputs were processed in Perseus (version 1.6.15.0) (69), and proteins identified as potential contaminants were removed. LFQ expression data were log_2_ transformed. Data were filtered to keep only those proteins with valid LFQ values in at least 70% of the samples in at least 1 of the sample groups, resulting in 3,371 protein groups annotated to 3,356 genes. Missing values were imputed for each sample on the basis of normal distribution to simulate low-abundance LFQ values. For the DDA dataset (*n* = 63), batch-effect correction was performed in Perseus via the R plugin using the limma package (72). Only samples from the proximal small intestine (*n* = 52) were included for downstream data analysis.

For the DIA dataset (*n* = 15), samples with valid LFQ values below the median minus 2 times the SD across all runs were excluded from downstream analysis (*n* = 3). Data were filtered to keep only proteins with valid LFQ values in at least 70% of the samples, resulting in 6,653 protein groups annotated to 6,594 genes across 12 samples. Biological pathways were annotated to proteins in Perseus, selecting GO biological processes, GO molecular function, GO cellular component, and KEGG.

### Proteomics data analysis and visualization

Global protein expression was compared between sample groups using an unpaired, 2-sided Student’s *t* test with permutation-based FDR control (FDR < 0.05; *n* = 250 randomizations). A *P* value of less than 0.01 was considered significant. Biological pathway annotation of proteins was exported from Perseus, and expression of proteins mapped to biological pathways were compared between groups by Mann-Whitney *U* test using Benjamini-Hochberg correction for multiple testing. All box plots show the median (center line) with the IQR (25%–75%), and whiskers show the furthest point within 1.5 times the interquartile length. Pathway enrichment analysis was performed by enabling categorical enrichment during PCA in Perseus (Benjamin-Hochberg–controlled FDR < 0.05). The resulting categories (enriched pathways), difference, and adjusted *P* values were exported, and proteins from the crypt datasets were mapped to the pathways. To compare mouse and human data, human gene names were converted to mouse gene orthologs using the “homologene” package in R followed by manual annotation of missing genes. To estimate the time- and IFN-γ dose–dependent change in protein expression, locally weighted regression (LOESS) smoothing was applied using the loess() function in R within the ggplot2 package. Default parameters were used, including a smoothing span of 0.75 and a polynomial degree of 2. CIs were estimated using standard errors derived from LOESS smoothing. The model was applied independently to each protein analyzed. As a measure of kinetics, we calculated the point of the curve at 50% change in expression comparing 0 and 9 IFN-γ injections. For human crypt proteome data, the median *z*-scored expression of proteins mapped to the GO biological pathway “response to interferon gamma” was calculated for each biopsy and correlated to published Vh/Cd ratio and biopsy proteome scores (Pearson’s correlation). Proteins from up- or downregulated biological processes in human crypts were subjected to protein-protein interaction network analysis using StringDB (73). Each node represents a protein, and each edge represents a physical or functional interaction between proteins. Networks were exported to Cytoscape 3.10.1 (74) and overlayed with a Student’s *t* test log_2_ difference from human crypts (UCeD vs. control) and mouse crypts (IFNx9 versus PBS) to annotate node size and node fill, respectively. Mouse orthologs for HLA molecules were manually assigned. Nodes were manually assigned to functional groups shown as gray regions. Data were plotted in R (version 4.2.3) using R Studio (build 421) and the ggplot2 package, and the final figures were assembled from PDF files in Adobe Illustrator.

### Immunofluorescence staining and detection of EdU in mouse small intestine

FFPE sections (3 μm) were deparaffinized and subjected to heat-induced antigen retrieval using high-pH antigen retrieval by incubation in preheated buffer in a water bath at 98°C for 20 minutes, followed by cooling for 20 minutes at room temperature. Sections were stained with monoclonal rabbit anti-Ki67 (clone SP6 from Dako or Abcam) or rabbit anti-lysozyme (Dako, EC 3.2.1.17) antibodies, followed by detection with goat anti–rabbit A488 (Molecular Probes, A-11034) or donkey anti–rabbit Cy3 (Jackson ImmunoResearch, 711-165-152) antibodies. Incorporated EdU was detected using the Click-iT EdU Cell Proliferation Kit for Imaging (Invitrogen, Thermo Fisher Scientific) according to the manufacturer’s instructions. Slides were counterstained with DAPI and mounted with ProLong Diamond Antifade Mountant (Thermo Fisher Scientific). Slides were imaged at room temperature on an inverted Nikon Eclipse Ti-S fluorescence microscope using a Nikon 9 10/0.3 or 9 20/0.45 Plan Fluor lens and Nikon NIS ELEMENTS BR 5.30.04 software. Images were processed in Fiji/ImageJ (2.9.0/1.53t) (75).

### Measurement of Vh/Cd ratios and EdU migration front

Measurement of Vh/Cd ratios was performed on immunofluorescence images stained for DAPI and Ki67. Crypt depth was measured from the crypt bottom to the end of the Ki67 signal. Villous height was measured from the end of the Ki67 signal to the tip of the villous as defined by DAPI signal. From each mouse, 3–5 segments from the proximal part of the intestine were analyzed (*n* = 3–6 villous-crypt pairs per segment). Average values for each mouse are reported. The EdU migration front was measured on immunofluorescence images stained for DAPI and EdU, measuring from the crypt bottom to the end of the EdU signal and reported in micrometers. Migration speed was calculated by dividing the distance from the migration front to the crypt bottom (in μm) by the time since EdU injection (22 hours). Values represent mean values for 5–7 segments (*n* = 3–5 crypts per segment) for each mouse. For C57Bl/6J mice injected with 9 doses IFN-γ and for *Ifngr1^IEC–/–^* littermate control mice injected with 6 doses IFN-γ, the EdU migration front had reached the end of the villi, and the calculated migration speed therefore represent the minimum migration velocity.

### Flow cytometric analysis of MHC II expression on gut epithelial cells

Biopsy specimens from the duodenum were collected from adult participants. Each participant donated 4–12 duodenal biopsy samples. The biopsy specimens were placed in ice-cold RPMI-1640 immediately after collection. The biopsies were treated twice with 2 mM EDTA in 2% FCS in PBS for 10 minutes at 37°C to release the IEC layer. Subsequently, the biopsies were finely chopped with surgical scissors and subjected to 2 rounds of digestion with 1 mg/mL collagenase type 4 (Worthington Biochemical) in 2% FCS in HBSS at 37°C for 45 minutes. During both the EDTA treatment and the pooled collagenase digestions, the samples were placed on rotation to ensure consistent exposure of the tissue to the reagents. After the first round of collagenase digestion, the samples were further homogenized using a 1 mL pipette and filtered through a 40 μm cell strainer to obtain a suspension of lamina propria cells. Remaining undigested pieces were treated with buffer containing fresh collagenase for a second round of digestion. Single-cell suspensions of the 2 EDTA fractions and the pooled fractions from collagenase treatment were subsequently used for flow cytometric analysis. We used a 24-color antibody panel (Supplemental Table 9) to stain the tissue samples for 30 minutes on ice with a mix of antibodies including a LIVE/DEAD marker. This panel included the monoclonal antibodies SPV-L3 (anti–HLA-DQ pan) and 2.12.E11 (anti–HLA-DQ2), which on HLA-DQ2 molecules recognize nonoverlapping epitopes (76). The samples of the 3 single-cell suspension fractions were analyzed on a Sony ID7000 spectral flow cytometer at the Flow Cytometry Core Facility at Oslo University Hospital. Autofluorescence correction is a unique feature in spectral flow cytometry and was applied to all acquired samples. Fluorescence minus one (FMO) controls for the HLA-DQ–reactive antibodies were undertaken for all 3 cell fractions. The flow cytometric data were analyzed with FlowJo software (version 10.10.0).

### Statistics

Statistical analysis was performed in Persues or in R (version 4.2.3) using R Studio (build 421). Protein expression was compared between 2 groups using an unpaired, 2-sided Student’s *t* test with permutation-based FDR control (FDR < 0.05; *n* = 250 randomizations). A *P* value of less than 0.01 was considered significant. Pathway enrichment analysis was performed by enabling categorical enrichment during PCA in Perseus (Benjamin-Hochberg–controlled FDR < 0.05). Expression of proteins mapped to biological pathways were compared between groups by Mann-Whitney *U* test using Benjamini-Hochberg correction for multiple testing. *P* values are reported in each figure legend, where a *P* value of less than 0.05 was considered significant. Figures display box plots that show the median (center line) with the IQR (25%–75%), and whiskers show the furthest point within 1.5 times the interquartile length. Pearson’s correlation coefficient (*R*) and associated *P* values were computed for scatter plots that also show the fitted linear regression lines and 95% CIs.

### Study approval

All participants provided written informed consent to donate material to research prior to participation in this study. The gut biopsies were part of the Institutional Research Biobank (no. 20521), and use of the material was approved by the Norwegian Regional Committee for Medical and Health Research Ethics South-East (REK Sør-Øst; approval no. 6544). All mouse experiments were approved by the Norwegian Food Safety Authority (Mattilsynet) (FOTS 22198, 30621).

### Data availability

Mass spectrometry proteomics data have been deposited to the ProteomeXchange Consortium via the PRIDE (77) partner repository with the dataset identifiers PXD062610 and PXD062684. Data from the study can be found in the Supplemental Figures and Supplemental Tables, and values for data points in Figure graphs are reported in the Supporting Data Values file. Any additional information required to reanalyze the data reported in this work is available from the corresponding authors upon request.

## Author contributions

J Stamnaes designed the research, performed tissue proteomics and immunofluorescence experiments, analyzed the data, and wrote the manuscript. DS performed tissue proteomic experiments and data analysis on human samples. MFDP and AED designed and performed mouse experiments. LFR designed and analyzed flow cytometry experiments on human gut biopsies. J Shaik performed flow cytometry experiments. MS performed proteomics analysis. KEAL was responsible for biobanking of human samples. LMS designed the research, wrote the manuscript, and supervised the project. All authors approved the final manuscript.

## Supplementary Material

Supplemental data

Supplemental table 1

Supplemental table 2

Supplemental table 3

Supplemental table 4

Supplemental table 5

Supplemental table 6

Supplemental table 7

Supplemental table 8

Supplemental table 9

Supporting data values

## Version 1 08/19/2025

In-Press Preview

## Version 2 10/15/2025

Electronic publication
