# Supplementary material for: IFN-γ is a direct driver of crypt hyperplasia in celiac disease
Source: J Clin Invest. 2025 Aug 19;135(20):e194858. doi: 10.1172/JCI194858 (PMC12520675; doi:10.1172/JCI194858)
Supplement: Supplemental data [file jci-135-194858-s077.pdf]

Supplemental figures and tables for

Interferon-γ is a direct driver of crypt hyperplasia in celiac disease

Jorunn Stamnaes[1,2,3*], Daniel Stray[1], M. Fleur du Pré[1,3], Louise F. Risnes[1,4], Alisa E. Dewan[1,3], Jakeer Shaik[3], Maria Stensland[2], Knut E. A. Lundin[1,4], Ludvig M. Sollid[1,3 *]

[1] Norwegian Coeliac Disease Research Centre, Institute of Clinical Medicine, University of Oslo, Oslo, Norway

[2] Proteomics Core Facility, University of Oslo and Oslo University Hospital, Oslo, Norway

[3] Department of Immunology, Oslo University Hospital-Rikshospitalet, Oslo, Norway

[4] Department of Gastroenterology, Oslo University Hospital-Rikshospitalet, Oslo, Norway

[*] Correspondence to:

Ludvig M. Sollid

l.m.sollid@medisin.uio.no

Jorunn Stamnaes

jorunn.stamnas@medisin.uio.no

Department of Immunology, Oslo University Hospital-Rikshospitalet, NO-0372, Oslo, Norway

P: +47 23073811

F: +47 23073510

This supplement contains:

Supplemental Figures S1-S9

Supplemental Tables as separate .xlsx files

Supplemental Table 1 (separate .xlsx file)

Demographics of FFPE duodenal biopsy samples used for crypt proteome analysis

Supplemental Table 2 (separate .xlsx file)

Proteins quantified from human crypt samples

Supplemental Table 3 (separate .xlsx file)

Two-sample Student's *t* test comparison of proteins from human crypt samples

Supplemental Table 4 (separate .xlsx file)

Biological pathways enriched along PC1 in human crypts

Supplemental Table 5 (separate .xlsx file)

Demographics of fresh duodenal biopsy samples used for flow cytometry analysis

Supplemental Table 6 (separate .xlsx file)

Proteins quantified from mouse crypt samples of C57BL/6 mice treated with IFN-ɣ

Supplemental Table 7 (separate .xlsx file)

Enriched biological pathways along PC1 from mouse crypt samples (shared proteins)

Supplemental Table 8 (separate .xlsx file)

Proteins quantified from mouse crypt samples (*Ifngr1* $^{IEC-/-}$ and controls)

Supplemental Table 9 (separate .xlsx file)

24-color antibody panel for SONY ID7000 flow cytometry analysis

Supplemental Figure 1

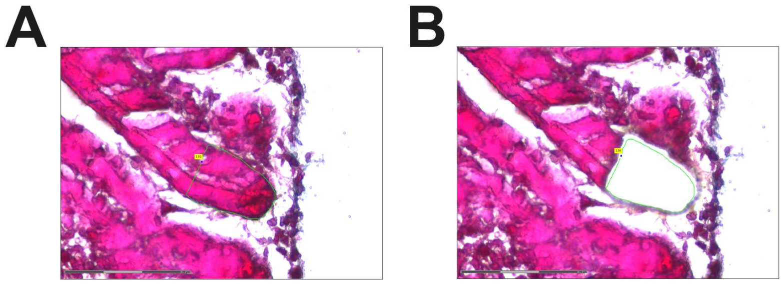

Supplemental Figure 1. Isolation of crypt regions by laser capture microdissection. Identification of crypt region isolated for proteome analysis in human small intestinal tissue based on the presence of eosinophilic Paneth cell granules, before (**A**) and after (**B**) LCM capture. Scale bar 75µm.

Supplemental Figure 2

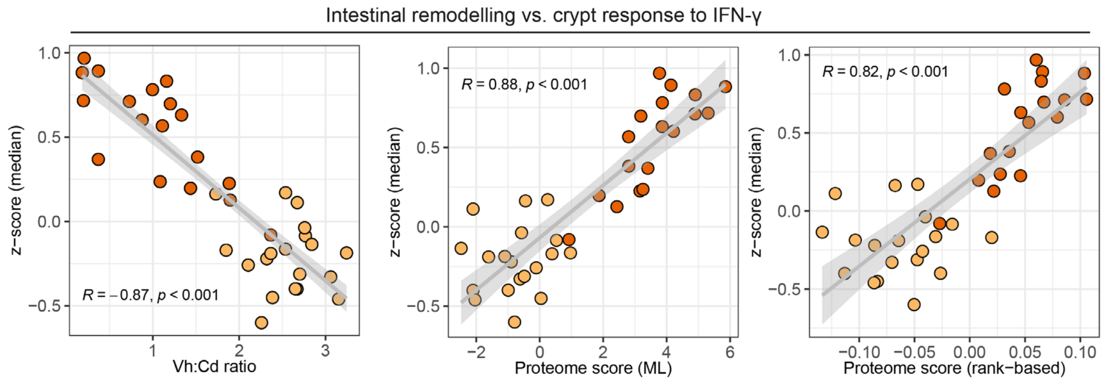

Supplemental Figure 2. Correlations between intestinal remodeling and crypt IFN-γ response in CeD. Correlation between intestinal remodeling measured by Vh:Cd ratio and biopsy proteome scores (ML score based on logistic regression and rank-based scores (1)) and median z-scored expression of proteins that map to Gene Ontology Biological Pathway "Response to interferon gamma" in crypt samples from untreated CeD (dark orange) and treated CeD (orange) biopsies. Each data point represents one biopsy block. R = Pearson's correlation coefficient.

Supplemental Figure 3

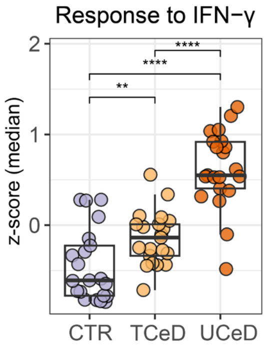

Supplemental Figure 3. Expression of IFN-γ response proteins in villous epithelium. Proteins from LCM-isolated villous epithelium mapped to GSEA Hallmark «Response to interferon gamma» filtered on presence in the human CeD crypt proteome dataset (n = 30). Each data point represents one protein and shows median z-scored expression per group. *P* values determined using Mann-Whitney U test with Benjamin Hochberg correction for multiple testing. * *P* < 0.05, ** *P* < 0.01, *** *P* < 0.001, **** *P* <0.0001, ns: non-significant. (UCeD, untreated CeD; TCeD, treated CeD; CTR, non-CeD control).

Supplemental Figure 4

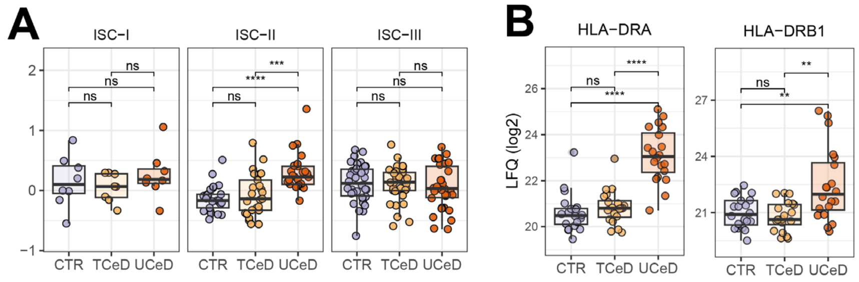

Supplemental Figure 4. Crypt protein expression of ISC markers and HLA-DR. (**A**) Z-scored expression of proteins mapped to ISC subtype genesets from (2) (ISC-I n = 8; ISC-II n = 25; ISC-II n = 32). Each data point represents one protein and shows median z-scored expression per group. (**B**) Log2 LFQ-protein expression of HLA-DRA and HLA-DRB1 protein in human crypt samples. Each data point represents one biopsy sample. (**A-B**) *P* values determined using Mann-Whitney U test with Benjamin-Hochberg correction for multiple testing. * $P < 0.05$, ** $P < 0.01$, *** $P < 0.001$, **** $P < 0.0001$, ns: non-significant.

Supplemental Figure 5

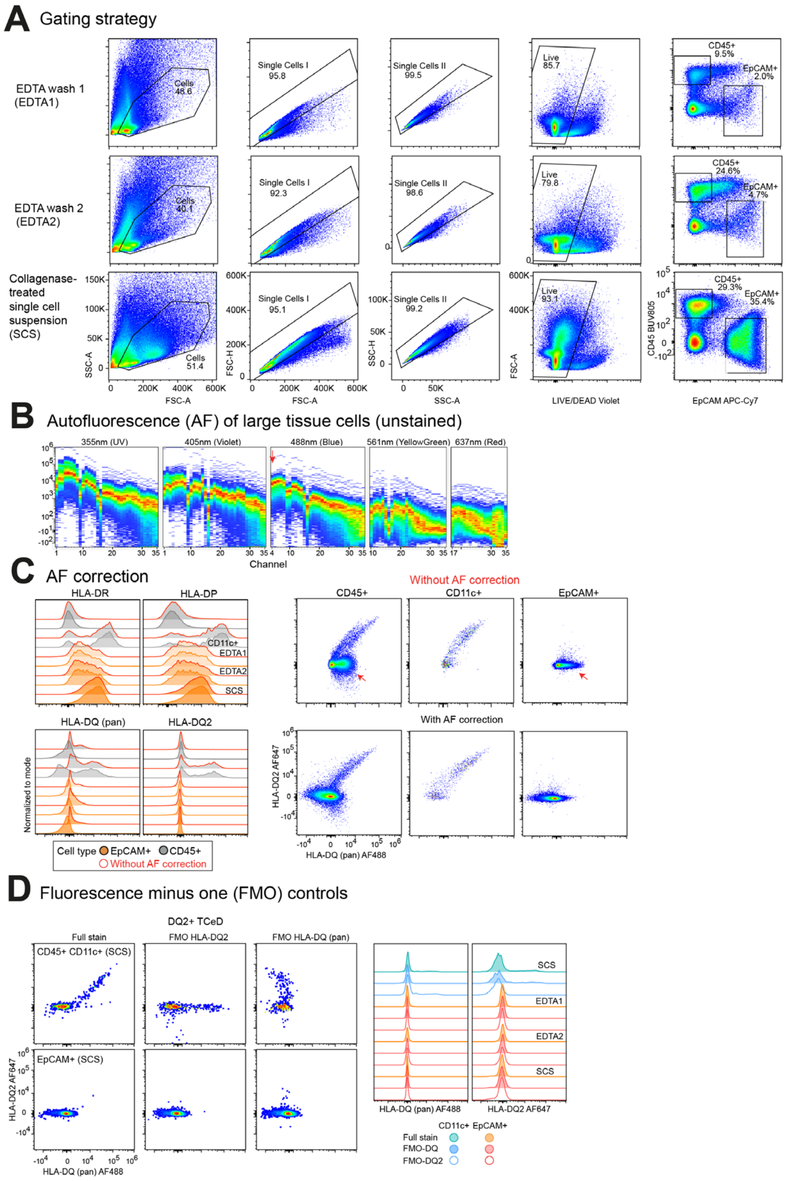

Supplemental Figure 5. Flow cytometry analysis of gut epithelial cells. (**A**) Gating strategy illustrated with representative forward scatter plots, side scatter plots and viability (live/dead) plots as well plots depicting staining for CD45 and EpCAM of three cell fractions of an untreated CeD patient, the same subject as in Figure 3A. (**B)** Spectral signature of autofluorescence (AF) across all lasers from unstained large tissue cells. (**C**) AF correction was applied to the samples and had most impact on AlexaFluor488 channel used for labelling of the anti-HLA-DQ (pan) antibody (indicated with red arrows) (**D**) Fluorescence minus one (FMO) controls for the anti-HLA-DQ2 and anti-HLA-DQ-pan antibodies are shown for a DQ2+ TCeD patient.

Supplemental Figure 6

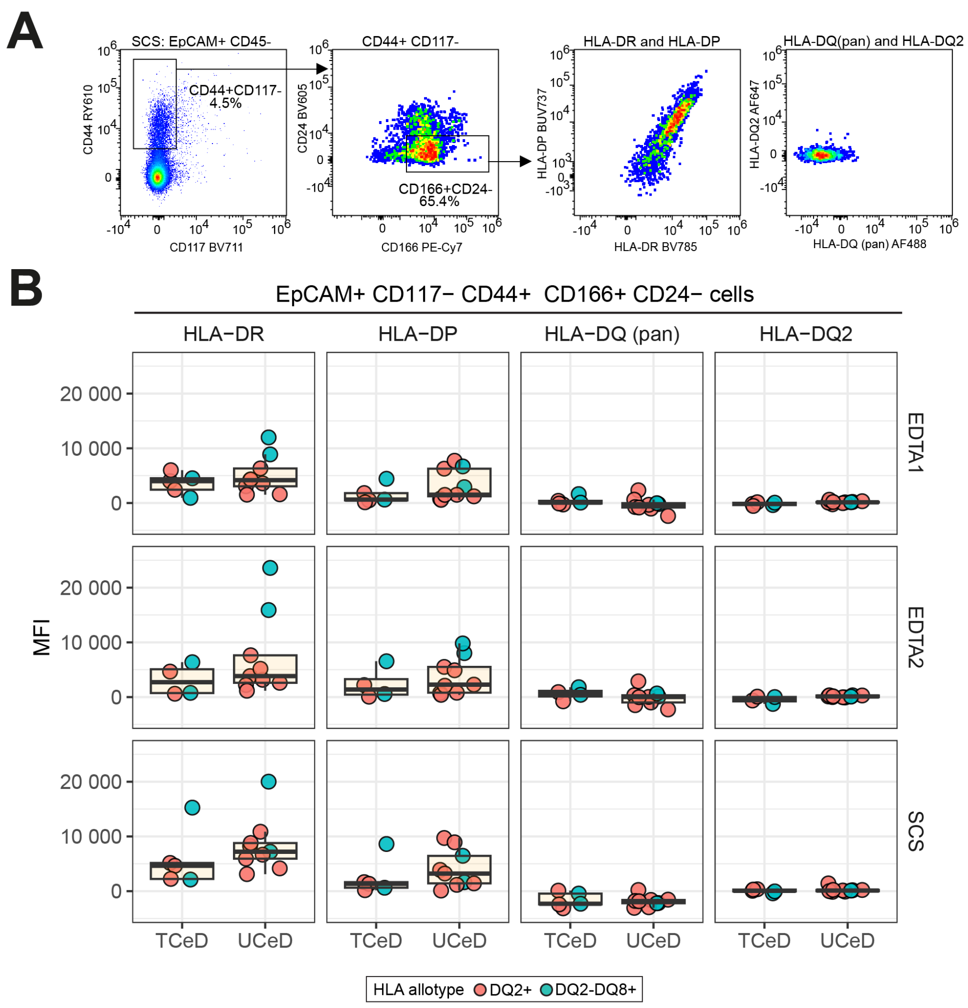

Supplemental Figure 6. HLA expression on gut epithelial cells enriched for crypt cells. (**A**) Flow cytometry gating strategy for EpCAM+ CD45- epithelial cells of a SCS fraction being CD44+ CD117- CD166+ CD24-, the same subject as in Figure 3A. Cells with this phenotype should encompass ISCs. Staining for HLA-DR, HLA-DP, HLA-DQ (pan) and HLA-DQ2 is shown. (**B**) Staining results of the three fractions EDTA1, EDTA2 and SCS from 9 UCeD and 5 TCeD subjects given as median fluorescence intensity (MFI).

Supplemental Figure 7

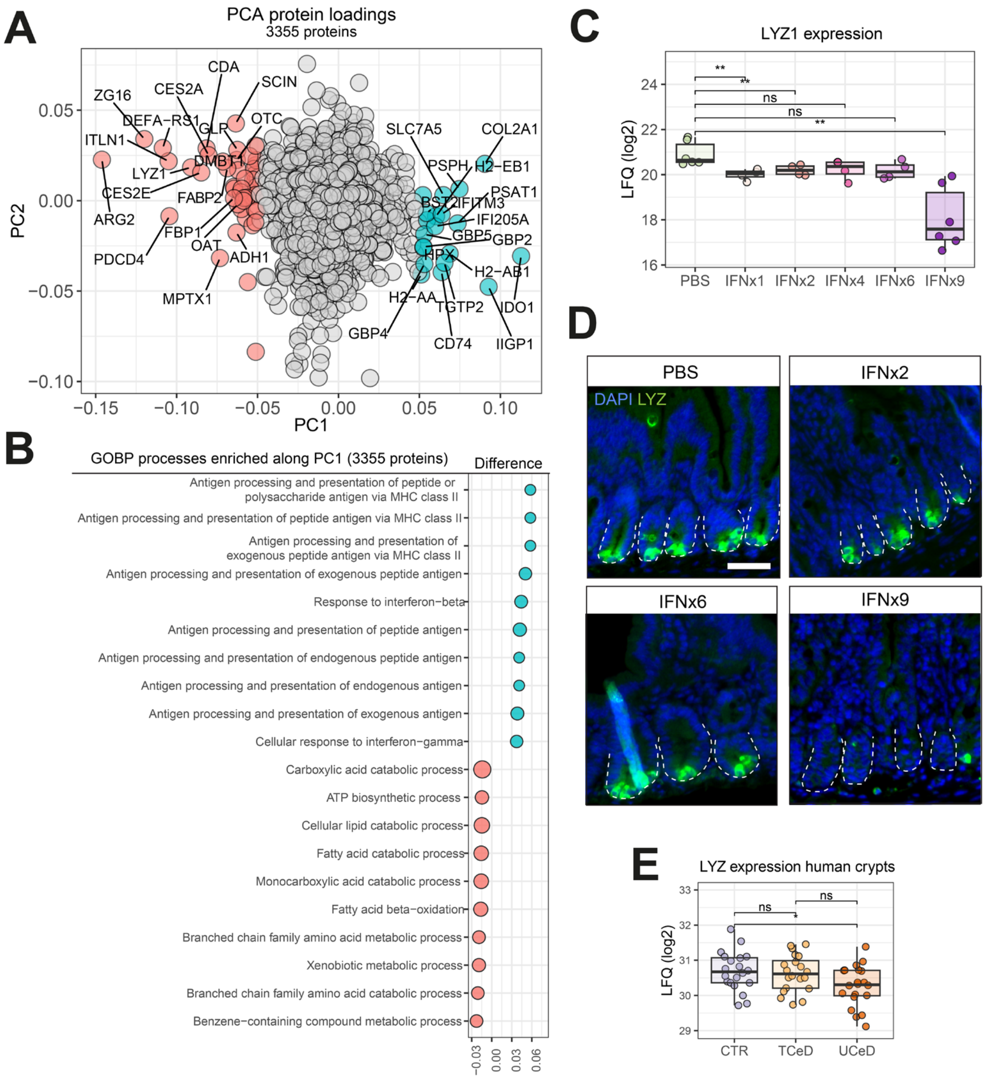

Supplemental Figure 7. IFN-γ induces changes in protein expression in the intestinal crypt region of mice. (**A**) Protein loadings driving separation of mouse crypt samples along PC1 and PC2 (Fig. 4F). (**B**) Top twenty enriched Gene Ontology Biological Processes along PC1 from (A). Pathways colored in blue are enriched in crypts following IFN-γ. (**C**), Log2 LFQ protein expression of LYZ1 in mouse crypts. Each datapoint reflects one mouse. (**D**) Representative images of LYZ staining (green) in mouse crypts. Nuclei are stained with DAPI (blue). Scale bar, 50 µm. (**E**) Log2 LFQ expression of LYZ protein in human crypt samples. Each dot represents one biopsy block. (**C, E**) *P* values determined using Mann-Whitney U test with Benjamin-Hochberg correction for multiple testing. * $P < 0.05$, ** $P < 0.01$, ns: non-significant.

Supplemental Figure 8

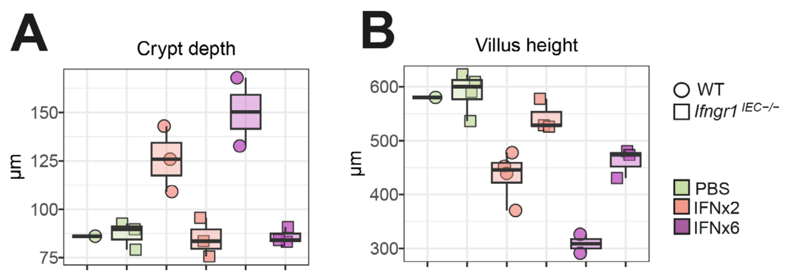

Supplemental Figure 8. Targeted deletion of *Ifngr1* in the epithelial cell prevents IFN-γ induced tissue remodeling. Measurement of crypt depth (**A**) and villus height (**B**) based on Ki67 staining and DAPI staining of nuclei. Each data point represents one mouse. Values represent median from 3-5 segments from the proximal small intestine (3-6 measurements per image).

Supplemental Figure 9

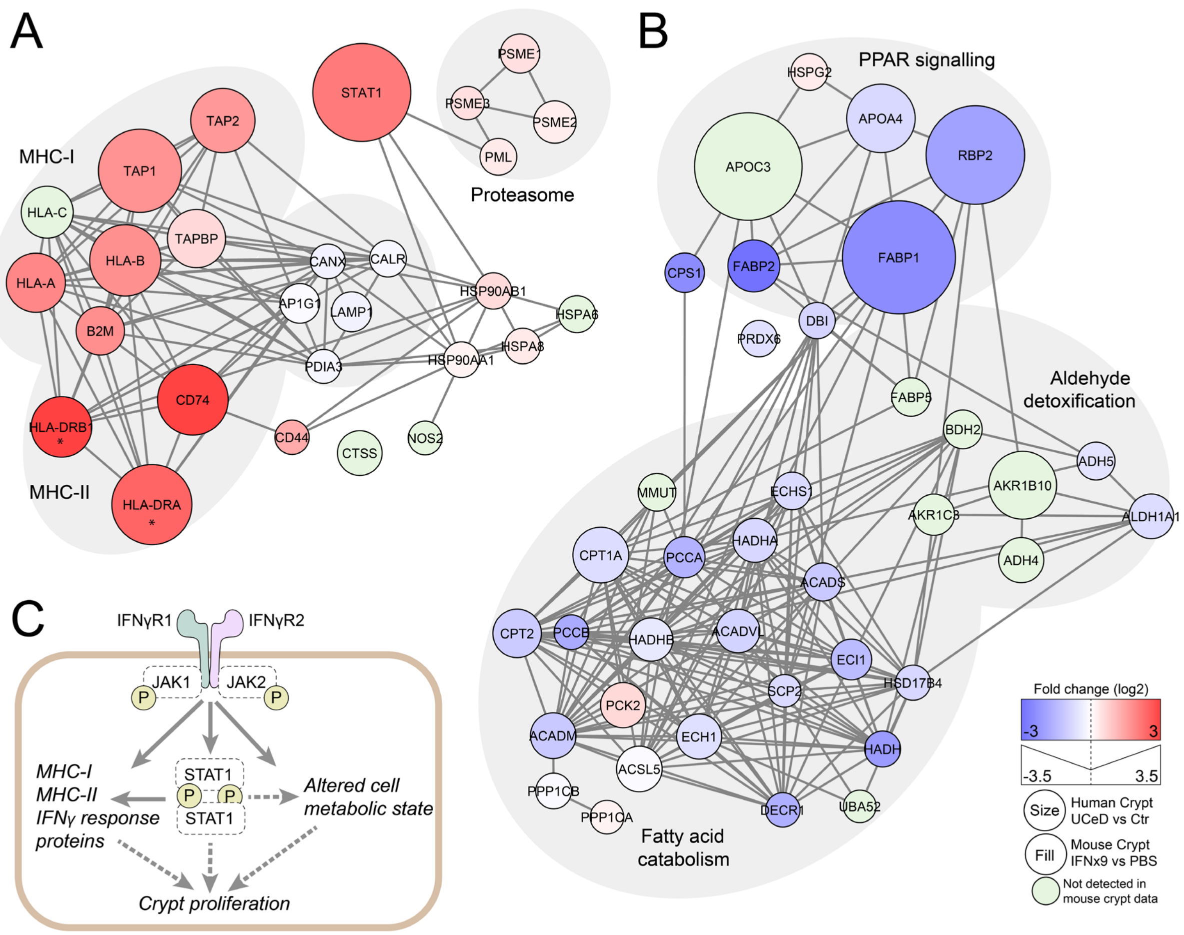

Supplemental Figure 9. Functional network of IFN-γ regulated proteins in UCeD crypts. StringDB protein-protein interaction network shows functional interaction between proteins mapped to regulated pathways that **(A)** increase (Figure 2D) or **(B)** decrease (Figure 2E) in UCeD crypts. Node size represents protein fold change in human crypts (log2 difference from Student's *t* test UCeD vs Ctr) and node fill represents fold change in mouse crypts (log2 difference from Student's *t* test IFNx9 vs PBS). Edges reflect physical (A) or (B) functional protein-protein interactions. *As C57BL/6 mice do not express the mouse HLA-DR homolog H2-EA, HLA-DRA and HLA-DRB have been mapped to H2-AA and H2-AB1, respectively. **(C)** Schematic representation of IFN-γR1 driven remodeling events in human celiac and mouse small intestine.

References

1. Johansen A, Sandve GKF, Ibsen JH, Lundin KEA, Sollid LM, and Stamnaes J. Biopsy proteome scoring to determine mucosal remodeling in celiac disease. *Gastroenterology.* 2024;167(3):493-504 e10.
2. Biton M, Haber AL, Rogel N, Burgin G, Beyaz S, Schnell A, et al. T helper cell cytokines modulate intestinal stem cell renewal and differentiation. *Cell.* 2018;175(5):1307-20 e22.
